# A Novel Co_3_O_4_@Co_3_(HITP)_2_‐Based Sensor for Low‐Temperatures H_2_S Detection: Fabrication, Performance, and Preliminary Exploration in Monitoring Pork Spoilage

**DOI:** 10.1002/advs.202521071

**Published:** 2026-01-15

**Authors:** Yongjiao Sun, Rongrong He, Wuchao Tian, Bingliang Wang, Zhenting Zhao, Zihan Wei, Koichi Suematsu, Wendong Zhang, Kengo Shimanoe, Jie Hu

**Affiliations:** ^1^ Center of Micro/Nano Devices and Intelligent Sensing College of Electronic Information Engineering Taiyuan University of Technology Taiyuan Shanxi P. R. China; ^2^ Laboratory of Electronic Functional Materials Huizhou University Huizhou P. R. China; ^3^ Department of Physics School of Physical and Mathematical Sciences Nanjing Tech University Nanjing P. R. China; ^4^ Department of Advanced Materials Science and Engineering Faculty of Engineering Sciences Kyushu University Kasuga Fukuoka Japan

**Keywords:** Co_3_(HITP)_2_, Co_3_O_4_, H_2_S detection, low‐temperature gas sensor, meat‐spoilage monitoring

## Abstract

Conductive metal–organic frameworks (cMOFs) are promising for room‐temperature gas sensing, yet their development for highly sensitive detection at refrigeration temperature remains a challenge. This work addresses this gap by developing a heterostructured Co_3_O_4_@Co_3_(HITP)_2_ chemiresistor for hydrogen sulfide (H_2_S) sensing. The decoration of Co_3_(HITP)_2_ with Co_3_O_4_ nanoparticles facilitates carrier transfer and lowers the activation energy for H_2_S reaction. The optimized sensor demonstrates notable sensitivity, with response values (*R_g_/R_a_*) of 3.0 and 1.4 toward 10 ppm H_2_S at 25°C and 40°C, respectively. The excellent low‐temperature performance is primarily attributed to the Co_3_(HITP)_2_. As a proof‐of‐concept, a portable device with a smartphone interface was employed to monitor H_2_S released from pork spoilage at both temperatures. It is important to note that early spoilage involves complex volatile profiles, and sole reliance on H_2_S detection may be limited due to potential temporal mismatches with other key spoilage markers. This study presents a functionalization strategy to extend cMOF‐based sensing into lower temperature regimes, offering a material platform for chilled meat safety monitoring while acknowledging the constraints of single‐analyte detection for early warning.

## Graphical abstract

Hybrid Co_3_O_4_@Co_3_(HITP)_2_‐based chemiresitor was constructed for hydrogen sulfide detection at low‐temperature, which presented excellent sensing performance at room and refrigerant temperature. A proof of concept was conducted by monitoring hydrogen sulfide released from pork spoilage at both temperature using a portable device with a smartphone interface.

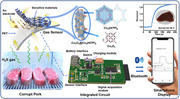

## 1 Introduction

Hydrogen sulfide (H_2_S) is a highly toxic, flammable acidic gas posing significant risks to human health and industrial safety. Even at low concentrations, H_2_S is toxic to the human body, while inhaling high concentrations can be rapidly fatal through respiratory paralysis and systemic cytotoxicity [1, 2]. However, H_2_S is an indispensable raw material in the fields of industrial production and environmental protection [3]. Besides, as the storage time increases, microorganisms will decompose the sulfoprotein, causing the meat to spoil and produce foul‐smelling and unpleasant H_2_S even in a refrigerating environment [4, 5, 6]. Therefore, the ability to promptly detect H_2_S is crucial to ensuring the well‐being of individuals and food safety. Conventional H_2_S sensors often require elevated operating temperatures to achieve optimal detection performance, which conflicts with the growing demand for low‐power sensing system [7]. Moreover, the necessity for thermal activation introduces inherent safety risks due to the combustible nature of H_2_S, potentially leading to hazardous scenarios during in situ monitoring. The detection of H_2_S instantaneously and reliably remains challenging due to the low sensitivity and selectivity, irreversibility, and instability of the sensor at low temperature.

To achieve the portable electronics for H_2_S monitoring, it is essential to minimize working temperature and improve sensitivity and selectivity, which is hard to achieve with conventional metal oxides [8]. Among the various materials employed in room‐temperature gas sensors, notable materials include graphene, transition‐metal dichalcogenides (TMDs), carbon nanotubes (CNTs), polyaniline (PANi), polypyrrole (PPy), MXene, and conductive metal–organic frameworks (cMOFs) [9, 10, 11, 12, 13, 14]. cMOFs, in particular, have garnered significant attention due to their high specific surface area, abundant pores and adsorption sites, and moderate electrical conductivity [15]. Co_3_(HITP)_2_ with exceptional selectivity and reversibility, stands out as the most useful cMOFs for H_2_S sensing [16]. Nevertheless, Co_3_(HITP)_2_‐based gas sensor still faces challenges, such as low sensitivity and inadequate stability, which restricts the commercialization of portable devices. To overcome the limitations of Co_3_(HITP)_2_‐based gas sensors, various approaches have been investigated, such as noble metal and classical metal oxide decoration [16, 17], and graphene oxide (GO) assembling [18]. Our group fabricated noble metal (Pd, Au, Pt) and SnO_2_ nanoparticles decorated Co_3_(HITP)_2_ sensor for improving the performance of H_2_S detection at room‐temperature [16, 17]. Xu et al. developed a room‐temperature NO*_x_* gas sensor using GO assembling Co_3_(HITP)_2_ [18]. While various nanomaterials decorations have been reported for enhancing the sensing performance of Co_3_(HITP)_2_, the functionalization of p‐type metal oxides and sensing at room or lower temperature has not been widely explored yet.

**SCHEME 1:**
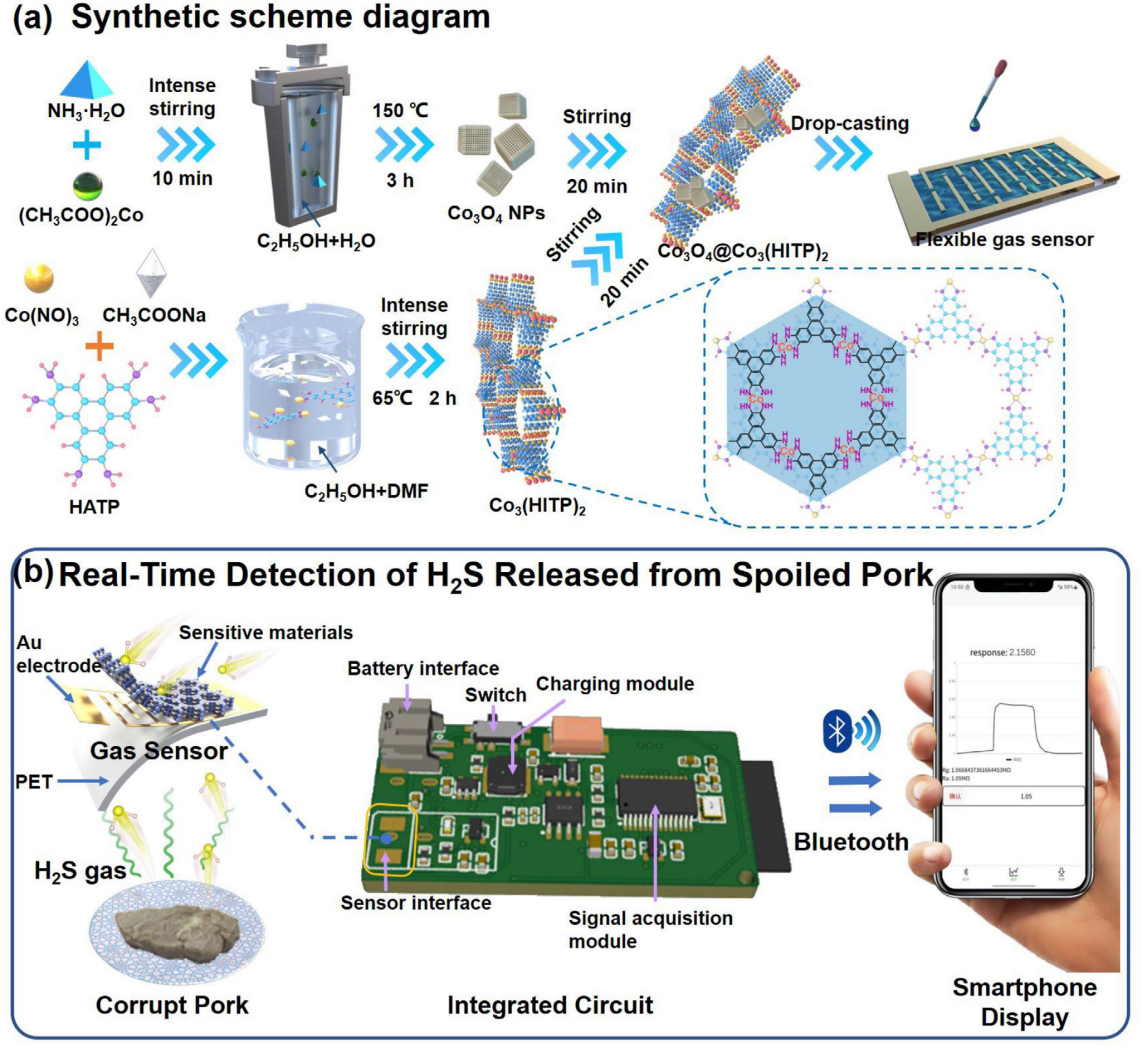
A fully integrated portable wireless H_2_S sensing system for real‐time monitoring of spoiled pork. (a) Schematic illustration of fabrication process for Co_3_O_4_@Co_3_(HITP)_2_ gas sensor. (b) Overview of the portable sensing system incorporating a Co_3_O_4_@Co_3_(HITP)_2_‐based sensor and a wireless communication system.

Herein, we designed and synthesized Co_3_O_4_ modified Co_3_(HITP)_2_‐based chemiresistive sensors with high response and selectivity toward H_2_S at room and refrigerating temperature (0°C–4°C), demonstrating outstanding property at low temperature compared with conventional chemiresistors. Smaller size and appropriate modification of Co_3_O_4_ leads to better sensing performance. Co_5_‐12@Co_3_(HITP)_2_ exhibits exceptionally high response of 3.0 and 1.4 toward 10 ppm toward H_2_S at room and refrigerating temperature with a low theoretical detection limit calculated to be sub‐ppb level. After combining with circuitry and Bluetooth transmission technology, the developed portable device is then applied in an exploratory test to monitor pork freshness stored at both room and refrigerating temperatures (Scheme 1). This preliminary demonstration successfully verified the ability of Co_5_‐12@Co_3_(HITP)_2_ sensor to detect H_2_S under these relevant conditions, highlighting its potential contribution to non‐invasive monitoring in low‐temperature environments.

## 2 Results and Discussion

### 2.1 Characterization

The process of Co_3_O_4_@Co_3_(HITP)_2_ synthesis and the program of fabricating a gas sensor is illustrated in Scheme 1a. Co_3_O_4_@Co_3_(HITP)_2_ was synthesized by using solvothermal method and transferred directly upon the flexible Au interdigital electrode to make a gas sensor. The portable wireless real‐time H_2_S detection system for spoiled pork monitoring introduced here consists of a Co_3_O_4_@Co_3_(HITP)_2_‐based sensor and a wireless communication system based on a Bluetooth low energy (BLE) chip, as described in Scheme 1b. The flexible chemiresistive gas sensor is connected to a BLE system‐on‐chip‐integrated circuits, with all components integrated on a rigid board. This integration enables real‐time monitoring of dynamic response through wireless communication and Android application‐based user interface. Additionally, all‐real‐time monitoring data will be recorded, and subsequently, the data can be utilized for food safety analysis purposes.

**FIGURE 1:**
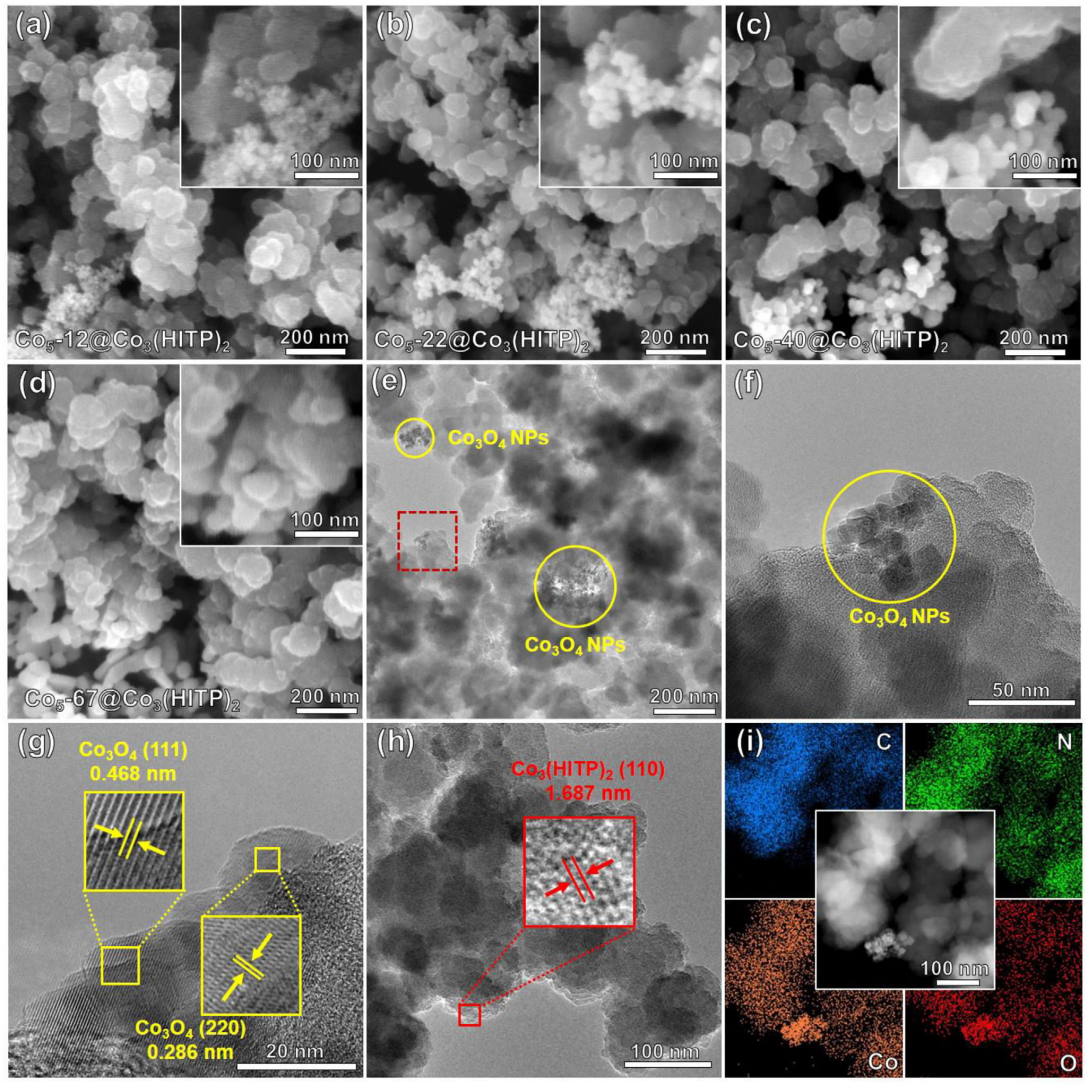
SEM images of Co_3_(HITP)_2_ loaded Co_3_O_4_ nanoparticles with different sizes of (a) 12 nm, (b) 22 nm, (c) 40 nm and (d) 67 nm. (e,f) TEM images of Co_5_‐12@Co_3_(HITP)_2_ at low and high magnification. (g,h) HRTEM images of Co_5_‐12@Co_3_(HITP)_2_. (i) HAADF‐STEM images and elemental mapping of Co_5_‐12@Co_3_(HITP)_2_.

The SEM images of as‐obtained Co_3_O_4_ nanoparticles with different sizes and their size distribution histograms (insets) are displayed in Figure S1. Co_3_O_4_ nanoparticles are uniformly and their average particle sizes (*P_s_*) are centered on 12, 22, 40, and 67 nm, respectively. Figure 1a–d show the SEM images of Co_3_O_4_ nanoparticles with different sizes decorated Co_3_(HITP)_2_ (Co_5_‐*x*@Co_3_(HITP)_2_, *x* = 12, 22, 40, and 67). The smaller Co_3_O_4_ nanoparticles agglomerate on the surface of larger Co_3_(HITP)_2_ particles. As shown in the TEM image for Co_5_‐12@Co_3_(HITP)_2_ in Figure 1e,f, the 12 nm Co_3_O_4_ nanoparticles gathered together in small groups disperse on the surface of Co_3_(HITP)_2_. HRTEM images in Figure 1g demonstrate that Co_3_O_4_ nanoparticles are highly crystalline and the gaps between lattice fringes are approximately 0.468 and 0.286 nm, which belong to the space of the (111) and (220) planes [19]. While, the low crystallinity of Co_3_(HITP)_2_ displays the hexagonal pores arranged in a honeycomb pattern with lattice fringes (Figure 1h) [20, 21, 22]. HAADF‐STEM and elemental mapping results (Figure 1i) indicate the widespread distribution of C, N, Co, and O elements on Co_5_‐12@Co_3_(HITP)_2_. Co atoms are well distributed across the entire heterostructure, while C, N, and O elements are clearly distinguishable in the separated areas, eventually confirming the fabrication of heterostructures. Figure S2a–e present the various amounts of 12 nm Co_3_O_4_ nanoparticles decorated Co_3_(HITP)_2_ (Co*_y_*‐12@Co_3_(HITP)_2_, *y* = 1, 2.5, 5, 10, and 20). The proportion of Co_3_O_4_ nanoparticles (12 nm) increases with the loading amounts. The EDS information of Co_5_‐12@Co_3_(HITP)_2_ further confirms the coexistence of C, N, Co and O in the sample (Figure S2f).

**FIGURE 2:**
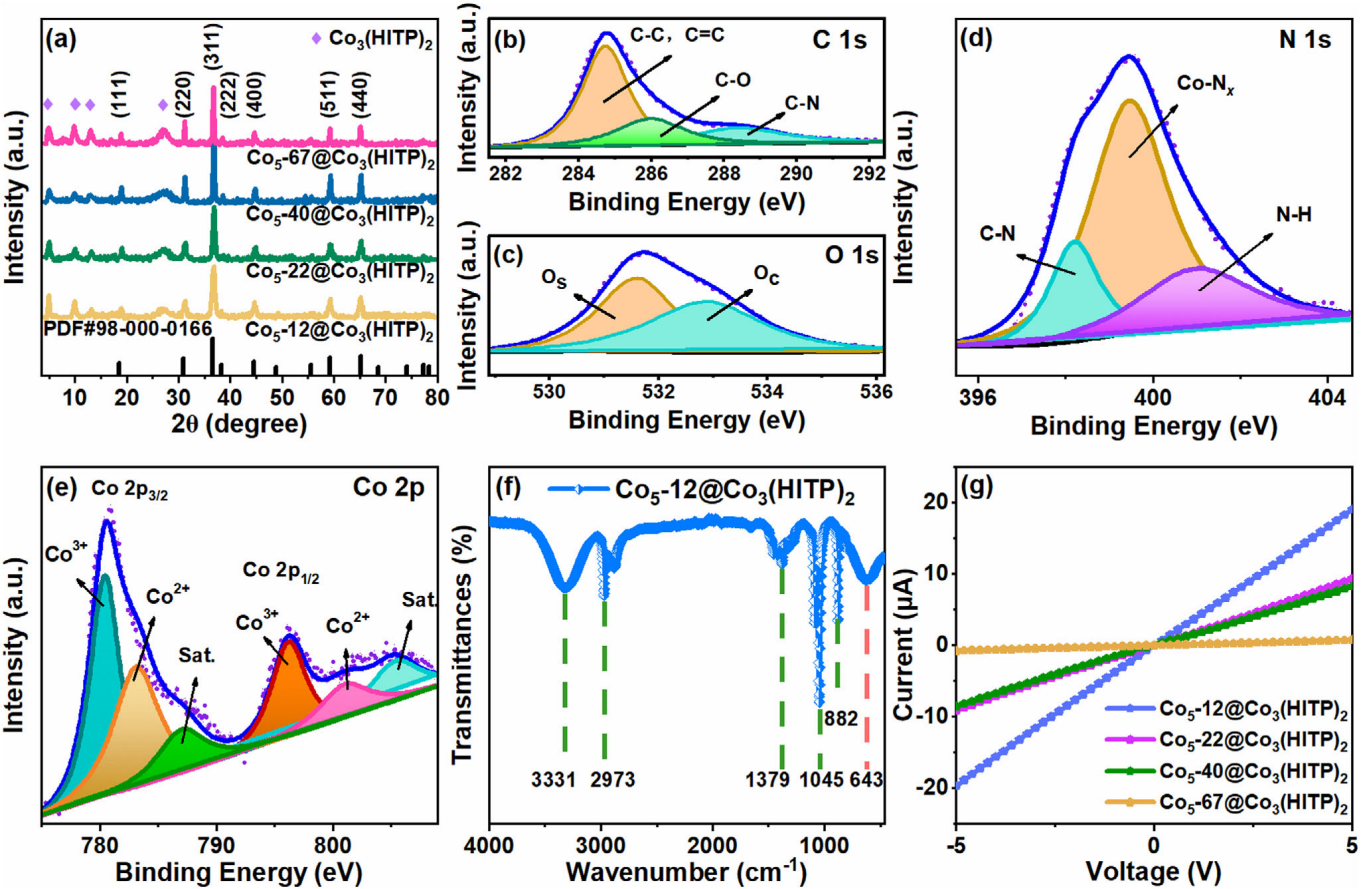
(a) XRD patterns of Co_5_‐*x*@Co_3_(HITP)_2_. XPS high‐resolution spectrum of Co_5_‐12@Co_3_(HITP)_2_: (b) C 1s, (c) O 1s, (d) N 1s, (e) Co 2p. (f) FTIR of Co_5_‐12@Co_3_(HITP)_2_. (g) *I*–*V* curve of Co_5_‐*X*@Co_3_(HITP)_2_ sensors in air atmosphere at room temperature.

To investigate the effect of different size of Co_3_O_4_ nanoparticles on the crystal structure, XRD patterns were employed to characterize the Co_5_‐*x*@Co_3_(HITP)_2_ (*x* = 12, 22, 40, and 67) samples (Figure 2a). The XRD patterns of all samples indicate characteristic peaks at 4.98°, 9.85°, 13.02°, 27.45°and 18.95°, 31.29°, 36.79°, 59.27°, 65.11°, which correspond to Co_3_(HITP)_2_ and (111), (220), (311), (511), (440) crystal planes (JCPDS: No. 42–1467) of typical spinel‐type Co_3_O_4_ [17, 23]. These peaks reveal the coexistence of Co_3_(HITP)_2_ and Co_3_O_4_ crystalline phases in all Co_5_‐*x*@Co_3_(HITP)_2_ samples. In addition, as the size of Co_3_O_4_ nanoparticles changes, the peak width at half height in XRD patterns also changes accordingly. Based on (220), (311), and (440) peaks of Co_3_O_4_, the crystallite size (D_s_) can be calculated using Debye Scherrer formula, and the corresponding results are listed in Table S1. Comparative analysis reveals significant differences in *D_s_* and *P_s_* for all Co_3_O_4_ samples except for the 12 nm Co_3_O_4_. This might be due to the agglomeration of grains to form larger nanoparticles.

The surface chemical composition of Co_5_‐12@Co_3_(HITP)_2_ was characterized by X‐ray photoelectron spectroscopy (XPS). Figure S3 shows the wide‐scan XPS spectrum of Co_5_‐12@Co_3_(HITP)_2_, revealing the presence of C, N, Co, and O elements. The C 1s spectrum consists of three peaks at binding energies of 284.3 eV, 286.0 eV, and 288.7 eV. These peaks correspond to the C─C/C═C, C─O, and C─N chemical bonds, respectively (Figure 2b) [17]. The high‐resolution XPS spectrum of O 1s in Figure 2c reveals two peaks at 531.5 eV and 532.8 eV, respectively, which are attributed to the surface oxygen species adsorbed over the oxygen vacancy (O_s_) and the chemisorbed oxygen (O_c_) [18, 24]. As shown in Figure 2d, the three peaks of N 1s at 398.1, 399.4, and 401.1 eV match well with the C─N, Co─N, and N─H bonds in the π‐conjugated groups, which are strongly associated with the formation of Co_3_(HITP)_2_ [21]. The high‐resolution Co 2p spectrum recorded by XPS presents two main spin‐orbit lines corresponding to Co^2+^ at 783.2 and 798.2 eV, and Co^3+^ at 780.4 and 796.1 eV (Figure 2e) [20, 25]. Meanwhile, the energy difference between the main peaks of the two different valence states is about 15.0 eV, which demonstrates the existence of Co_3_O_4_ [26].

Further information about the functional groups and chemical bonds of Co_5_‐12@Co_3_(HITP)_2_ were characterized using FTIR, as shown in Figure 2f. The absorption peaks around 1379 cm^−1^ could be attributed to the stretching vibration of the in‐plane stretching of the triplet phenylene ring, indicating the good‐in‐plane π‐conjugation [26]. Meanwhile, the absorption peaks near 1045 and 882 cm^−1^ are mainly ascribed to in‐plane bending vibrations of C─H bonds and in‐plane stretching vibrations of N─H bonds in the aromatic rings, respectively [21]. The peaks around 643 cm^−1^ corresponds to O─Co─O bond, which belong to Co_3_O_4_ [26, 27]. The broad absorption peak at 3331 cm^−1^ belongs to the ─OH stretching vibration of adsorbed H_2_O molecules. This also proves the existence of adsorbed H_2_O molecules on the surface of the sample prepared in the experiment, which is consistent with the results of O element distribution analysis [28].

Figure 2g and Figure S4 show the electronic features of Co_5_‐*x*@Co_3_(HITP)_2_ (*x* = 12, 22, 40, and 67) sensors by measuring *I*–*V* curves at different temperature in air atmosphere (two‐probe method). It can be seen that all of the sensors satisfy the linear characteristics, suggesting that the Au electrodes and the Co_5_‐*x*@Co_3_(HITP)_2_ are ohmic contact and no significant additional impedance is generated. With the Co_3_O_4_ nanoparticles size increase, the electroconductibility of the Co_5_‐*x*@Co_3_(HITP)_2_ decrease, which could be attributed to the reduced quantity of Co_3_O_4_ nanoparticles caused by larger size. Moreover, compared with the conductivity at room temperature, the conductivity of all sensors at higher temperature improves, which derives from the intrinsic property of semiconductor. From the Arrhenius‐type conductivity dependence in Figure S5, the thermal activation energy *E_a_* of the four sensors are calculated to be 0.0578, 0.0589, 0.0598, and 0.0871 eV, which possess low energy barriers for electron transition and facilitate the possible RT gas sensing properties [29].

### 2.2 Sensing Characteristics

**FIGURE 3:**
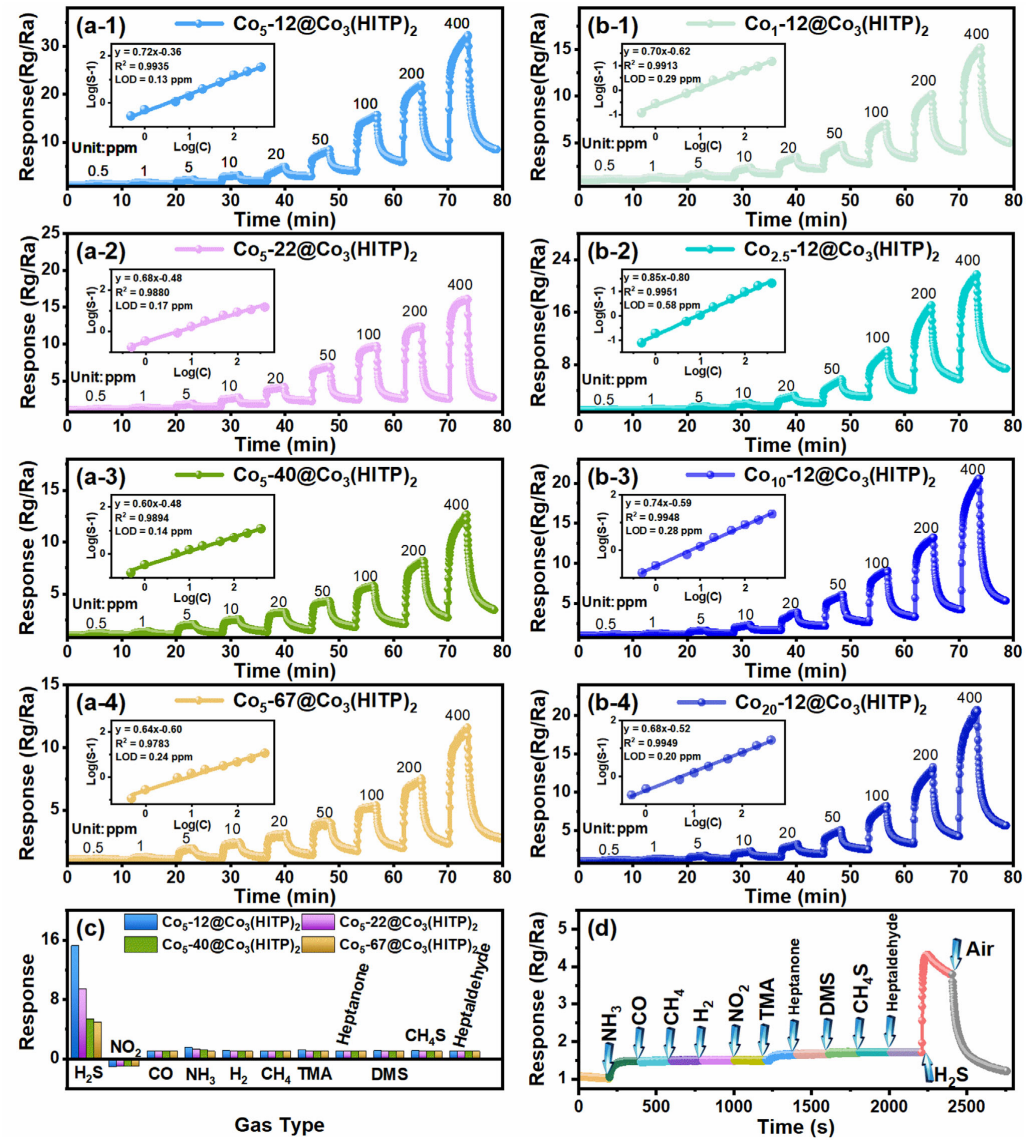
Dynamic response‐recovery curves of (a) Co_5_‐*x*@Co_3_(HITP)_2_ (*x* = 12, 22, 40, 67) and (b) Co*_y_*‐12@Co_3_(HITP)_2_ (*y* = 1, 2.5, 10, and 20) toward 0.5–400 ppm H_2_S under 25% RH at RT, insets: response‐concentration log‐log plots. (c) Response of Co_5_‐*x*@Co_3_(HITP)_2_ to 100 ppm different gases at RT. (d) Dynamic response‐recovery curves of Co_5_‐12@Co_3_(HITP)_2_ to 20 ppm H_2_S in the presence of 100 ppm interfering gases.

To assess the detection range and limit of all materials for H_2_S concentration, response value tests were conducted on all sensors toward 0.5–400 ppm H_2_S concentrations under 25% RH at RT. As shown in Figure 3a,b and Figure S6, the gas sensors based on Co_5_‐*x*@Co_3_(HITP)_2_ (*x* = 12, 22, 40, 67) and Co*_y_*‐12@Co_3_(HITP)_2_ (*y* = 1, 2.5, 10, and 20) exhibit sensitive and reversible response toward H_2_S at RT. In addition to the higher conductivity, which is consistent with the *I*–*V* results, all sensors present higher responses compared to the pure Co_3_(HITP)_2_ gas sensor. Besides, the gas sensor based on smaller Co_3_O_4_ nanoparticles decorated Co_3_(HITP)_2_ demonstrates a notably improved response compared with the other sensors, and the optimized amount of 5 wt.% for 12 nm Co_3_O_4_ nanoparticles decoration results in superior response values (Figure S7; Table 1) [16, 17]. It is believed that the smaller diameter of Co_3_O_4_ nanoparticles provides more active sites and higher catalytic activity for H_2_S gas adsorption and reaction [30]. Furthermore, only appropriate modification can achieve gas‐sensing sensitization effect, since excessive Co_3_O_4_ would actually hinder response improvement due to particle agglomeration [31]. Thus, the Co_5_‐12@Co_3_(HITP)_2_ sensor presents a response value as high as 3.0 at 10 ppm, and at H_2_S concentrations as low as 500 ppb, it still yields a response value of 1.28. The corresponding electric resistance in air of all sensors under 25% RH at RT are presented in Figure S8a. Moreover, the response values of Co_5_‐12@Co_3_(HITP)_2_ sensor exhibit an exceptional linear relationship with H_2_S concentration in logarithm form (inset), thus enabling us to estimate the H_2_S levels in practical applications accurately. The theoretical detection limit (LOD) of Co_5_‐12@Co_3_(HITP)_2_ sensor is predicted to be 130 ppb (set response equal to 1.1), highlighting the significant potential for application in H_2_S detection at RT.

**TABLE 1:** Response, increase response multiple to 100 ppm H_2_S and LOD of sensors in this work and our previous research.

Sample	Response (*Rg/Ra*)	Multiplier	LOD (ppb)	Refs.
Co_3_(HITP)_2_	4.3	1.0	975	[16]
Pd_2_‐Co_3_(HITP)_2_	8.1	2.0	213	[16]
Au_2_‐Co_3_(HITP)_2_	5.1	1.2	42	[16]
Pt_2_‐Co_3_(HITP)_2_	6.4	1.5	74	[16]
Sn_1_Co	5.5	1.3	322	[17]
Co_5_‐12@Co_3_(HITP)_2_	15.5	3.6	130	This work
Co_5_‐22@Co_3_(HITP)_2_	9.6	2.2	170	This work
Co_5_‐40@Co_3_(HITP)_2_	5.6	1.3	140	This work
Co_5_‐67@Co_3_(HITP)_2_	5.3	1.2	240	This work

The selectivity of Co_5_‐*x*@Co_3_(HITP)_2_ sensors were evaluated by measuring sensor response to 100 ppm H_2_S, NO_2_, CO, NH_3_, H_2_, CH_4_, TMA, heptanone, DMS, methanethiol, and heptaldehyde (Figure 3c). The response values toward H_2_S are over 5.3, while toward the other gases are below 1.5. Especially, the response of Co_5_‐12@Co_3_(HITP)_2_ sensor to H_2_S is 10.4 times higher than that to other gases, highlighting its exceptional selectivity toward H_2_S. The high selectivity to H_2_S originates from a robust coordination/redox interaction between the long pair electrons of H_2_S and the cobalt d‐orbitals, which yields chemical bonds that are significantly stronger and enables more efficient charge transfer compared to those formed with gases such as ammonia or carbon monoxide. To study the cross‐sensitivity of Co_5_‐12@Co_3_(HITP)_2_ sensor in a mixture of gases, the dynamic response to a mixed‐gas environment blending 100 ppm NH_3_, CO, CH_4_, H_2_, NO_2_, TMA, heptanone, DMS, methanethiol, heptaldehyde, and 20 ppm H_2_S were tested. As shown in Figure 3d, the response is just little changed by the presence of 100 ppm NH_3_ (∼0.45) at first and 100 ppm DMS (∼0.11), while CO, CH_4_, H_2_, NO_2_, TMA, heptanone, methanethiol, and heptaldehyde almost had no influence to the response. Finally, the response to 20 ppm H_2_S in the mixture atmosphere is 4.6, which is comparable to that obtained in pure H_2_S atmosphere (4.8), suggesting its superior anti‐interference performance.

**FIGURE 4:**
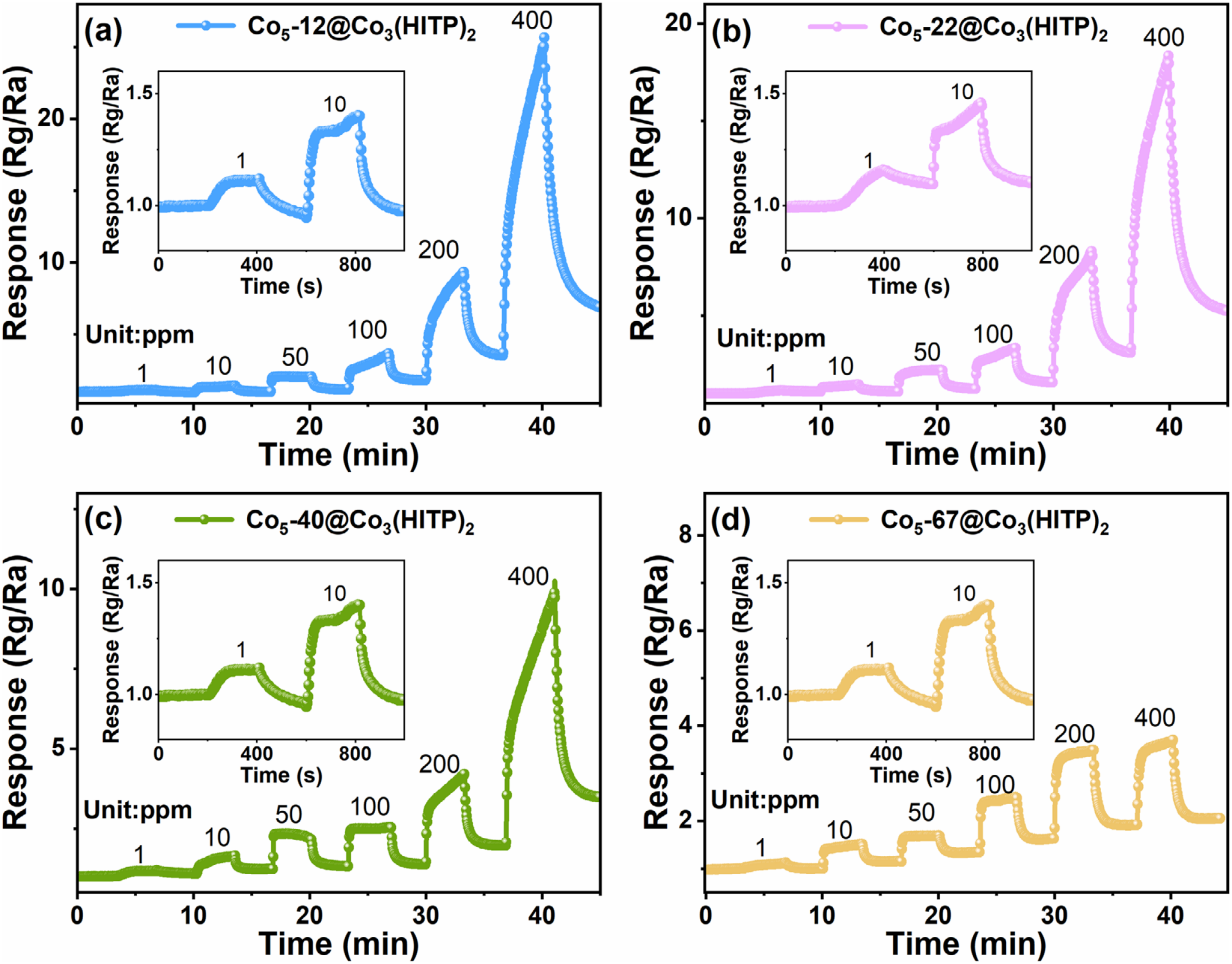
Dynamic response‐recovery curves of (a) Co_5_‐12@Co_3_(HITP)_2_, (b) Co_5_‐22@Co_3_(HITP)_2_, Co_5_‐40@Co_3_(HITP)_2_, and Co_5_‐67@Co_3_(HITP)_2_ toward 1–400 ppm H_2_S under 25% RH at low temperature (0°C–4°C).

Universally, the response of a semiconductor sensor will be changed with the working temperature and shows a higher response value at a certain working temperature, so the gas‐sensing performance at RT to 100°C were studied. We found that the Co_5_‐12@Co_3_(HITP)_2_ sensor demonstrates optimal sensing performance at RT (Figure S9), likely due to high‐temperature weaken interaction between H_2_S and sensing material [32], enabling accurate H_2_S detection in scenarios where sensor heating is impractical, such as natural gas fields and oil extraction sites. This represents a significant advantage over traditional MOS gas sensor, which exhibits notable limitation in such operating conditions. Based on the above findings, we anticipate that the proposed sensor is likely to be able to maintain well performance even at refrigerating temperature (0°C–4°C). The Co_5_‐*x*@Co_3_(HITP)_2_ sensors were placed in a refrigerating temperature environment, and their dynamic response‐recovery curves exposed to 1–400 ppm H_2_S under 25% RH were investigated (Figure 4). Fortunately, the Co_5_‐*x*@Co_3_(HITP)_2_ sensors still show satisfactory response to H_2_S and Co_5_‐12@Co_3_(HITP)_2_ sensor shows response of about 1.1–1 ppm H_2_S. However, compared to measuring at RT, the sensing response decrease somewhat (20.7%) from 32.3 to 25.6 (400 ppm). Notwithstanding, these results conclusively demonstrate the functional viability of our Co_5_‐*x*@Co_3_(HITP)_2_ sensors in refrigerated environments. The sensing properties of Co_3_(HITP)_2_ sensor at refrigerating temperature (Figure S10) and Co_3_O_4_ sensor at RT and refrigerating temperature (Figure S11) were also tested. The response of Co_3_(HITP)_2_ toward H_2_S was little decreased at refrigerating temperature compared with at RT [17], but for Co_3_O_4_ sensor, the response at refrigerating temperature is strange, which indicates that the low‐temperature sensing response comes from Co_3_(HITP)_2_ to some extent. These results are consisted with the thermal activation energy (0.0782 and 0.1140 eV for Co_3_(HITP)_2_ and Co_3_O_4_) based on *I*–*V* curves at different temperatures (Figures S12 and S13).

Considering the inherent sensitivity of semiconductor materials to humidity and the actual service conditions, the response of Co_5_‐12@Co_3_(HITP)_2_ sensor under different RH conditions was further evaluated. The results in Figure S14a–e indicate that as RH increases from 25% to 95%, the response to H_2_S drops dramatically. The declining quantity for 1 ppm and 200 ppm is 3.1% and 22.9% from 25% RH to 75% RH, while 7.3% and 60.8% from 25% RH to 95% RH, respectively (Figure S14f). Scrupulously, the influence of humidity on the response is relatively minor at lower RH levels, but becomes significantly greater at higher levels, which is in line with the trend of the resistance changes (Figure S15). A notable issue is that the effect of humidity intensifies as the detected H_2_S concentration increases, which might be relevant with the lower response value at lower concentration. To address the stability issues arising from humidity, subsequent work necessitates incorporating hydrophobic breathable membranes or relevant functional groups to enhance the sensor's moisture resistance.

### 2.3 Gas Sensing Mechanism

**FIGURE 5:**
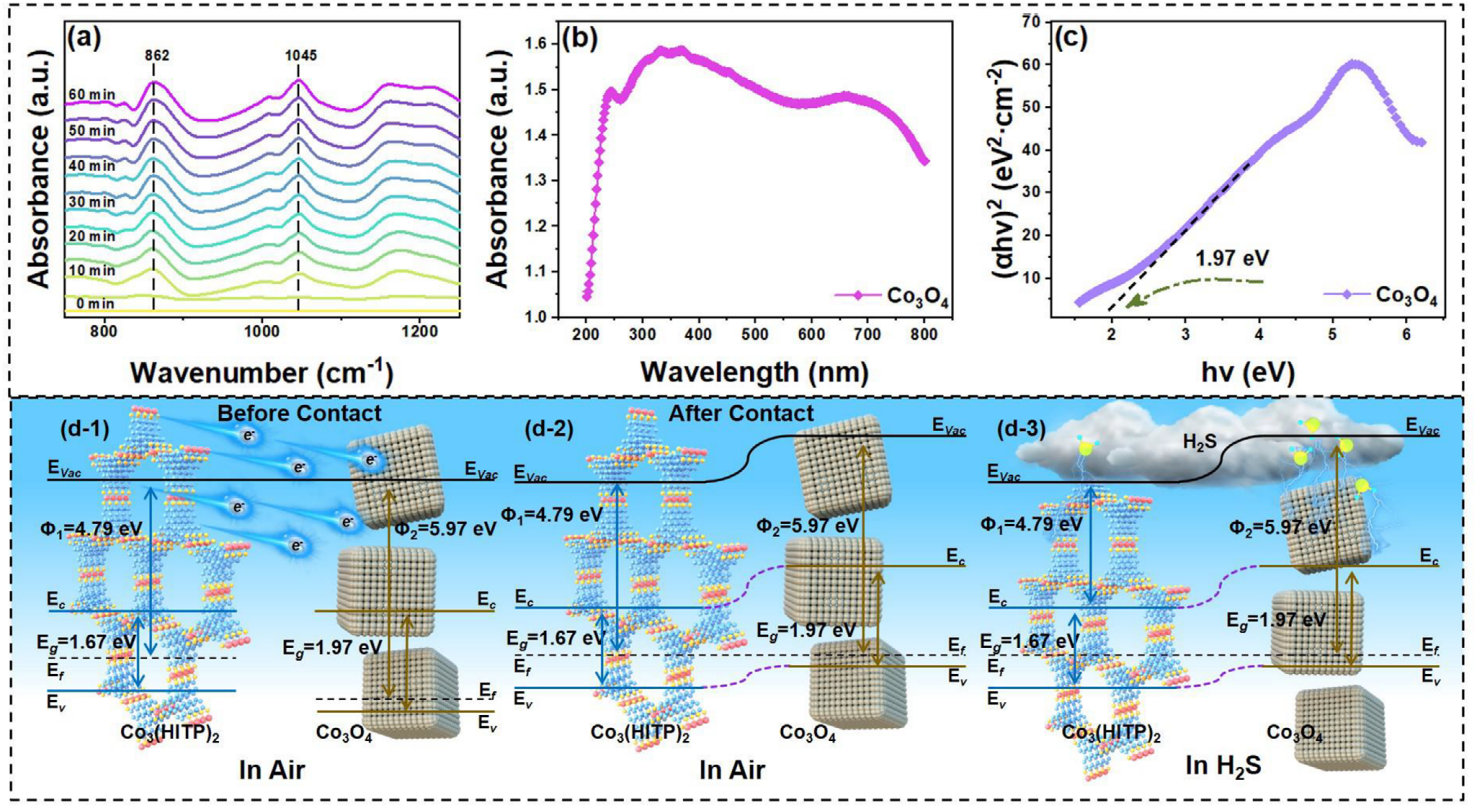
(a) Time‐resolved in situ DRIFTS of H_2_S absorption at RT on Co_5_‐12@Co_3_(HITP)_2_. (b) UV–vis spectra and (c) (ahν)^2^ vs. (hν) curve of Co_3_O_4_. (d) Schematic diagrams for energy band of Co_3_O_4_@Co_3_(HITP)_2_ composite before and after exposure to H_2_S.

We exposed Co_5_‐12@Co_3_(HITP)_2_ sensor to 10 ppm H_2_S (in air) for 60 min, followed by a period of purging the sensor with air, while monitoring the process by DRIFTS. Upon addition of H_2_S to the DRIFTS chamber, difference spectra reveal two main new spectral features (Figure 5a). The absorption peak at 862 cm^−1^ corresponds to the stretching vibration of N─H, while the absorption peak at 1045 cm^−1^ may correspond to the complex sulfate formed during the H_2_S adsorption process [33, 34]. As similar with our previous work, the sulfate might be produced by catalytic oxidation of H_2_S by Co node and cause the electron transfer from H_2_S to Co_3_(HITP)_2_ [17]. These results demonstrate that Co_5_‐12@Co_3_(HITP)_2_ has undergone adsorption with gas molecules in H_2_S gas environment, which involves charge transfer and results in change in the resistance of sensitive material.

The optical absorption properties of the Co_3_O_4_ nanoparticles (12 nm) were investigated at RT by UV–vis spectroscopy. Figure 5b shows the absorbance spectrum of Co_3_O_4_ nanoparticles (12 nm) with three absorption bands. The absorption bands (λ∼664 nm and λ<500 nm) indicate ligand‐metal charge transfer events O^2−^→Co^3+^ and O^2−^→Co^2+^, respectively [35, 36]. The absorption bandgap energy *Eg* can be determined by the equation (*ahv*)^*n*^   =   *A*(*hv* − *E_g_*), where *hv* is the photo energy, *α* is the absorption coefficient, A is a constant relative to the material and *n* is either 2 for a direct transition or 1/2 for an indirect transition. Here, *n* is 0.5 for Co_3_O_4_ nanoparticles. The plot of (*αhν*)^2^ vs. *hv* is shown in Figure 5c. From the Tauc plot, the absorption bandgap (*Eg*) is estimated as 1.97 eV by extrapolating to (*αhν*)^2^ = 0 [37]. Furthermore, according to previous reports, the work function of Co_3_O_4_ is about 5.97 eV [38]. And the work function and bandgap width of Co_3_(HITP)_2_ are 4.79 and 1.67 eV, respectively, as measured in our previous work [16].

To further understand the gas sensing performance of the Co_3_O_4_ decorated Co_3_(HITP)_2_ composite, the sensing mechanism needs to be further analyzed using band theory. Figure 5d is a schematic diagram illustrating the sensing mechanism of Co_3_O_4_@Co_3_(HITP)_2_ composite. After physical contact, free electrons transfer from Co_3_(HITP)_2_ with a lower work function and Fermi level to Co_3_O_4_ with a higher work function and Fermi level at the contact interface, while holes move in the opposite direction. This redistribution continues until the Fermi levels of both materials reach equilibrium [39]. During this process, the holes concentration on both sides of the heterojunction interface changes, which leads to band bending. Finally, holes Co_3_(HITP)_2_ and electrons of Co_3_O_4_ are assembled, resulting in a drop of resistance in Co_3_(HITP)_2_, which is consistent with *I*–*V* curves. The concentrated electrons of Co_3_O_4_ are in favor for the adsorption of oxygen ions and then lower the reaction activation energy [40]. When exposed to H_2_S, except for the directly electrons transferring from H_2_S to Co_3_(HITP)_2_, Co_3_O_4_ nanoparticles provides more active sites, which enables H_2_S adsorption and more electrons transfer to Co_3_O_4_@Co_3_(HITP)_2_ composite. This breaks the previously established equilibrium state, causing further increase in resistance, leading to an enhanced H_2_S sensing performance.

### 2.4 Detection of H_2_S in Spoiled Pork

**FIGURE 6:**
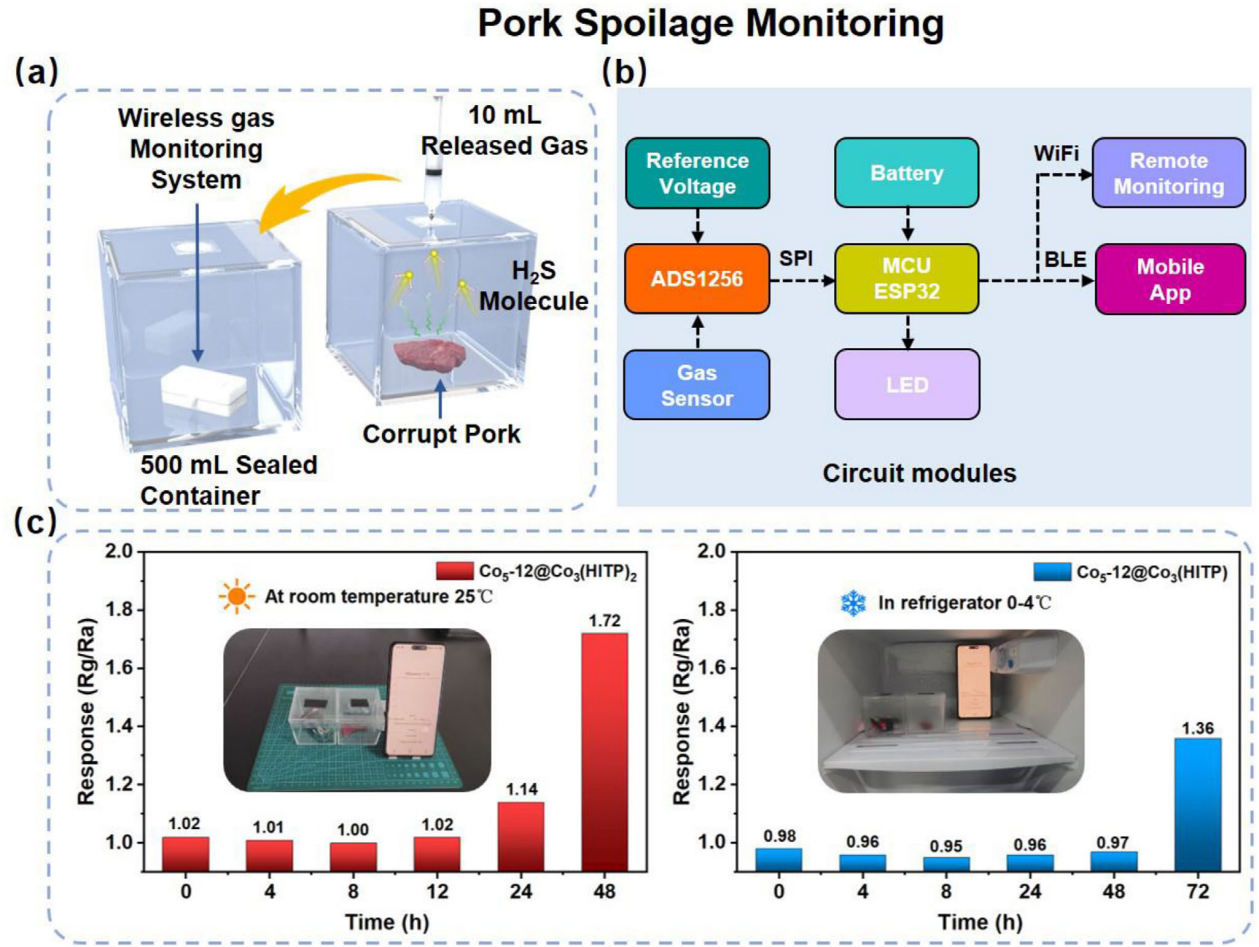
(a) A schematic diagram of Co_5_‐12@Co_3_(HITP)_2_ sensor for exhaled H_2_S detection. (b) The functional modules of the wireless gas monitoring system. (c) Response values of pork stored for different time at room and refrigerating temperature.

We developed a rigid printed circuit board (PCB) detector for monitoring the H_2_S released from spoilage pork detection as a proof‐of‐concept demonstration. This detector device primarily consists of several modules, as illustrated in Figure 6b. The data is transferred from the detector to the mobile phone through Bluetooth or to the terminal through WiFi. The rigid PCB detector is composed of a microcontroller unit (MCU) module, an analog‐to‐digital converter (ADC), a gas sensor, a type C connection module, and a battery (800 mAh) (Figure S17). The collected voltage signals are transmitted to the main controller via SPI communication. The detailed circuit diagram has been provided in Figure S18. The MCU master control module circuit integrates with Bluetooth and WiFi module. The analog front‐end circuit is responsible for signal conditioning and buffering. By using a voltage divider resistor and follower circuit, the sensor can output an appropriate ADC sampling voltage within a specified range. The power supply module is responsible for charging the lithium battery, adapting the voltage, and regulating the power. The low‐power consumption characteristic of the circuit provides a stable and long‐lasting power supply for the system. Figure S19 presents the software interface of the mobile phone application. On the screen, we can see the response change curves and the instantaneous resistance value of the detector along with the corresponding time.

The pork spoilage detection at room (25°C) and refrigerating (4°C) temperatures was operated to study the spoilage differences stored under different conditions. The contaminated pork was placed in a sealed container (500 mL), and during the storage, metabolic gases including H_2_S were filled into the container. Then, 10 mL gas extracted from the container of pork was injected into another same sealed container with our portable device every 4 h. The optical images of a piece of pork at room temperature and in refrigerator are shown in Figures S20 and S21. Figure 6a displays the schematic diagram for testing at refrigerating temperature in refrigerator. Two pieces of similar pork were stored in sealed containers indoor or in refrigerator, respectively. Figure 6c shows the response curves of the detector for pork stored at different temperatures for varying periods (several hours). The fresh or early‐stage pork spoilage produce little H_2_S and has small response. Starting from the second day, the response under room temperature increases from 1.02 to 1.14, and after stored for two days under room temperature, the response reaches 1.72. In refrigerator, the response come to 1.36 after stored for 3 days. Both the measurements under room and refrigerating temperature were recorded in Video S1. The pork stored in the refrigerator appears to spoil more slowly compared to store at room temperature over the same storage time. However, the actual evolution of H_2_S concentration relative to other spoilage volatiles in the container has not yet been confirmed by gas chromatography‐mass spectrometry (GC‐MS). Therefore, these results establish a correlation between pork spoilage and storage time, though they are limited in detection early spoilage.

## 3 Conclusion

In summary, we have developed a novel low‐temperature H_2_S sensor based on Co_3_O_4_‐decorated Co_3_(HITP)_2_. By optimizing the size and loading of Co_3_O_4_ nanoparticles, we achieved a sensor (with 5% loading, 12 nm Co_3_O_4_) that exhibits outstanding response and high selectivity toward H_2_S at both room and refrigeration temperatures. Band theory analysis reveals that the heterojunctions between Co_3_O_4_ and Co_3_(HITP)_2_ facilitates enhanced interaction with H_2_S molecules, thereby significantly improving sensing performance. As a preliminary and exploration, the Co_5_‐12@Co_3_(HITP)_2_ sensor was integrated with a wireless PCB platform to enable real‐time, selective monitoring of H_2_S released from spoiled pork under both room‐temperature and refrigerated conditions. However, it is important to explicitly note the limitation of using H_2_S as a sole indicator for early‐stage spoilage detection: in the actual complex decay process, H_2_S concentration may fall below the sensor's detection threshold or may not fully represent the meat's freshness status. In conclusion, this study focuses on the development and mechanism of a novel low‐temperature H_2_S sensor, with pork spoilage application serving as a proof‐of‐concept demonstration.

## Conflicts of Interest

The authors declare no conflicts of interest.

## Supporting information




**Supporting File**: advs73861‐sup‐0001‐SuppMat.docx.

**Supporting File**: advs73861‐sup‐0001‐SuppMat.docx.

## Data Availability

The data that support the findings of this study are available from the corresponding author upon reasonable request.
