# Supplementary material for: A Novel Co3O4@Co3(HITP)2‐Based Sensor for Low‐Temperatures H2S Detection: Fabrication, Performance, and Preliminary Exploration in Monitoring Pork Spoilage
Source: Adv Sci (Weinh). 2026 Jan 15;13(10):e21071. doi: 10.1002/advs.202521071 (PMC12915087; doi:10.1002/advs.202521071)
Supplement: Supplementary file 1 — Supporting File: advs73861‐sup‐0001‐SuppMat.docx. [file ADVS-13-e21071-s001.docx]

Supporting Information

**A Novel Co_3_O_4_@Co_3_(HITP)_2_-Based Sensor for Low-Temperatures H_2_S Detection: Fabrication, Performance, and Preliminary Exploration in Monitoring Pork Spoilage**

Yongjiao Sun^a^, Rongrong He^a^, Wuchao Tian^a^, Bingliang Wang^a^, Zhenting Zhao^b^, Zihan Wei^c^, Koichi Suematsu^d^, Wendong Zhang^a^, Kengo Shimanoe^d,*^, Jie Hu^a,*^

^a^Center of Micro/Nano Devices and Intelligent Sensing, College of Electronic Information Engineering, Taiyuan University of Technology, Taiyuan, Shanxi 030024, P. R. China

^b^Laboratory of Electronic Functional Materials, Huizhou University, Huizhou 516001 P. R. China

^c^Department of Physics, School of Physical and Mathematical Sciences, Nanjing Tech University, Nanjing 210009, P. R. China

^d^Department of Advanced Materials Science and Engineering, Faculty of Engineering Sciences, Kyushu University, Kasuga, Fukuoka 816-8580, Japan

**Corresponding authors:** Kengo Shimanoe, shimanoe.kengo.695@m.kyushu-u.ac.jp;

Jie Hu, [hujie@tyut.edu.cn](mailto:hujie@tyut.edu.cn);

1. **Experimental Section**

*Synthesis of Co_3_(HITP)_2_*: Co_3_(HITP)_2_ was synthesized through previously reported hydrothermal method.^[1]^ 27.1 mg of cobalt nitrate was dissolved in 1.5 ml of DMF, and preheated at 65℃. 8 mg of HITP was dissolved in 1.5 ml of deionized (DI) water. 0.328 g of anhydrous sodium acetate was dissolved in 2 ml of DI water. Then, HITP aqueous solution was firstly added to the cobalt nitrate solution, then sodium acetate aqueous solution was added to the above mixture. After that, the mixed solution was stirred at 65℃ in a water bath for fully reaction. The solution after reaction was naturally cooled to room temperature, and then centrifuged at a speed of 8000 r/s for 3 minutes using a high-speed freezing centrifuge. The black precipitate was obtained by washing with DI water and ethanol three times, respectively.

*Synthesis of Co_3_O_4_ particles*: Four kinds of size Co_3_O_4_ particles were synthesized.^[2]^ 0.5 g of cobalt acetate was dissolved in a mixture of 10 mL DI water and 15 mL of ethanol. The solution was vigorously stirred for 10 minutes, then 2.5 mL of concentrated ammonium hydroxide (14 mol/L) was added drop by drop and stirred for another 10 minutes. Afterwards, the above mixture was poured into a steel reactor and kept at 150℃ for 3 hours. After waiting for natural cooling, the obtained black Co_3_O_4_ (12 nm) was centrifuged and cleared with DI water and ethanol. The Co­_3_O_4_ nanoparticles size was changed through adjusting the ethanol amount in the solvent (the ratio of ethanol to water) or the concentration of cobalt acetate and ammonium hydroxide.

*Synthesis of Co_3_O_4_ Particles Decorated Co_3_(HITP)_2_*: Firstly, 5 mol% Co_3_O_4_ nanoparticles with different sizes were added to Co_3_(HITP)_2_ aqueous solution and stirred for 2 hours, then centrifugated with DI water and ethanol to obtain Co_3_O_4_@Co_3_(HITP)_2_. The samples were named as Co_5_-*x*@Co_3_(HITP)_2_ (*x* is the average size of Co_3_O_4_, *x*=12, 22, 40, and 67) for convenience. Secondly, y mol% 12 nm Co_3_O_4_ nanoparticles were decorated on Co_3_(HITP)_2_ using the same method and named as Co*_y_*-12@Co_3_(HITP)_2_ (*y*=1, 2.5, 5, 10, and 20).

*Material Characterizations*: Crystal phases were analyzed using XRD (Rigaku miniflex 600). Elemental chemical states were examined using XPS (Thermo ESCALAB250 Xi). Morphologies and structures were characterized using a SEM (Su8010, Hitachi) and TEM (JEM F200, JEOL). FTIR (TENSOR) and in situ DRIFTS were used to characterize the surface functional groups and chemical bonds. UV-vis spectrum was measured by using Agilent 8453 UV-vis spectrophotometer. A source meter (Keithley 2400) was used to measure the *I-V* curves of the fabricated sensors.

*Fabrication and Measurement of Co_3_O_4_ Particles Decorated Co_3_(HITP)_2_ Sensors*: 5 mg of Co_3_O_4_@Co_3_(HITP)_2­_ was dispersed in 1 mL of ethanol to form a homogeneous suspension. 2 μL of the above suspension was dropped into the surface of an Au interdigital electrode with a heating resistor on the back side. When dried naturally at room temperature, a gas sensor was fabricated. The sensing-performance of the as-prepared sensors were studied on an intelligent gas-sensitive analyzer (Elite CGS-4). Certain concentrations of target gases were prepared by using the static gas distribution method in a 1 L glass chamber. And the target gases balanced in high-purity air were sourced from a certified commercial gas cylinder (Taineng Gas Co., LTD). The temperature and relative humidity (RH) were adjusted through heating the resistor on the back side of the gas sensors and the saturated salt solutions. And the flexible characteristics were conducted on a flexible Au interdigital electrode without heating resistor. Since Co_3_O_4_@Co_3_(HITP)_2­_ sensors exhibited p-type sensing response, here we defined the sensing response *S* = *R_g_/R_a_*, (*R_g_* and *R_a_* were the resistances of the sensor exposed to reducing gases and air). Distinctively, for NO_2_, which is an oxidizing gas, the response *S* = *R_a_/R_g_*. The theoretical limit of detection (LOD) was calculated from the fitting line by set *S* = 1.1.

**2. Method of Calculation**

Debye Scherrer formula:

$\text{D=}\frac{\text{K}\text{λ}}{\text{β}\text{cos}\text{θ}}$ (1)

K≈0.9 gives an approximation of crystallite shape factor, λ is X-ray sources wavelength, β is full width at half maximum (FWHM), and θ is Braggsangle.^[3]^

**3. Materials Characterizations**


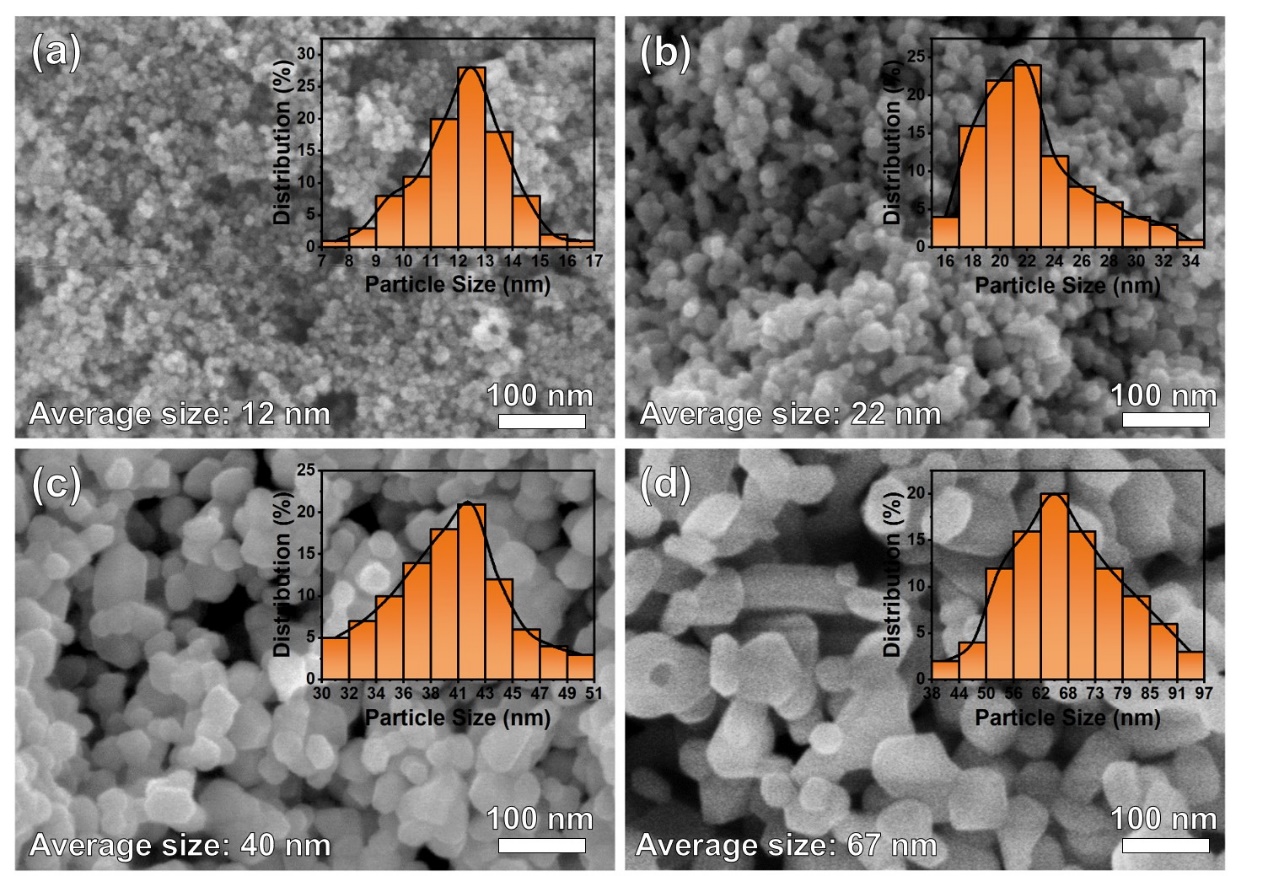


**Figure S1.** SEM images of Co_3_O_4_ nanoparticles with sizes of a) 12 nm, b) 22 nm, c) 40 nm and d) 67 nm. Insets show the distribution of Co_3_O_4_ nanoparticles sizes. The size of nanoparticles was obtained by calculating the average values of 100 particles, and the measuring error was lower than 10%.


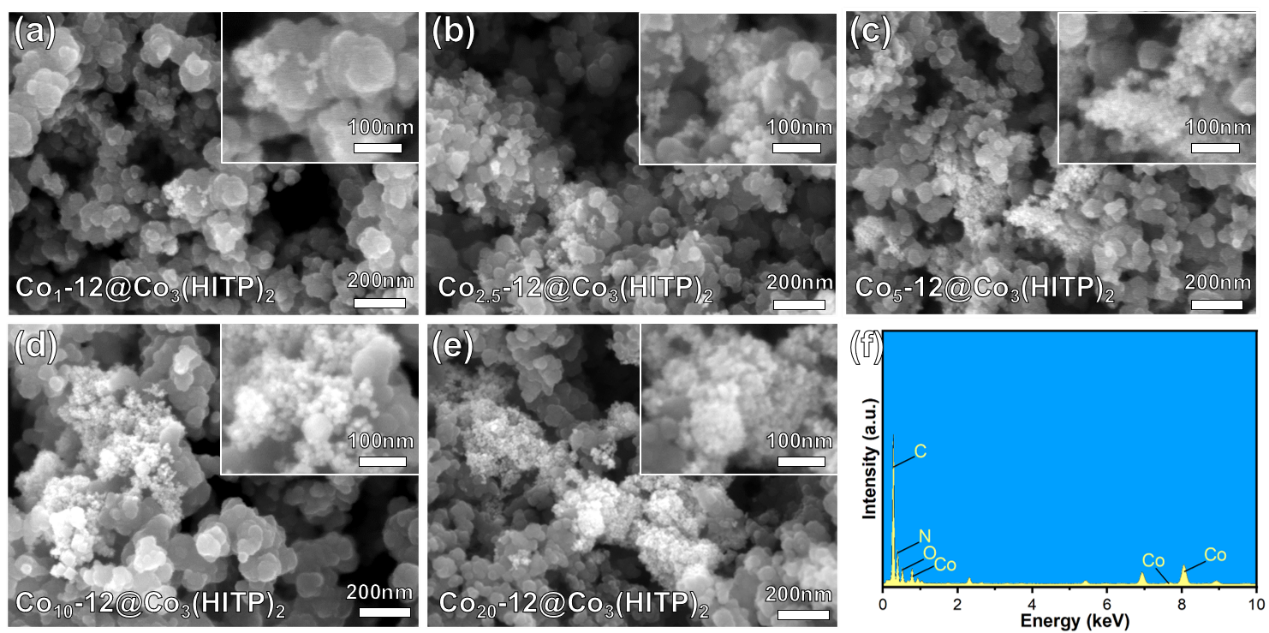


**Figure S2.** a–e) SEM images of Co_3_(HITP)_2_ loaded with different contents of 12 nm Co_3_O_4_ nanoparticles. f) EDS analysis of Co_5_-12@Co_3_(HITP)_2_.


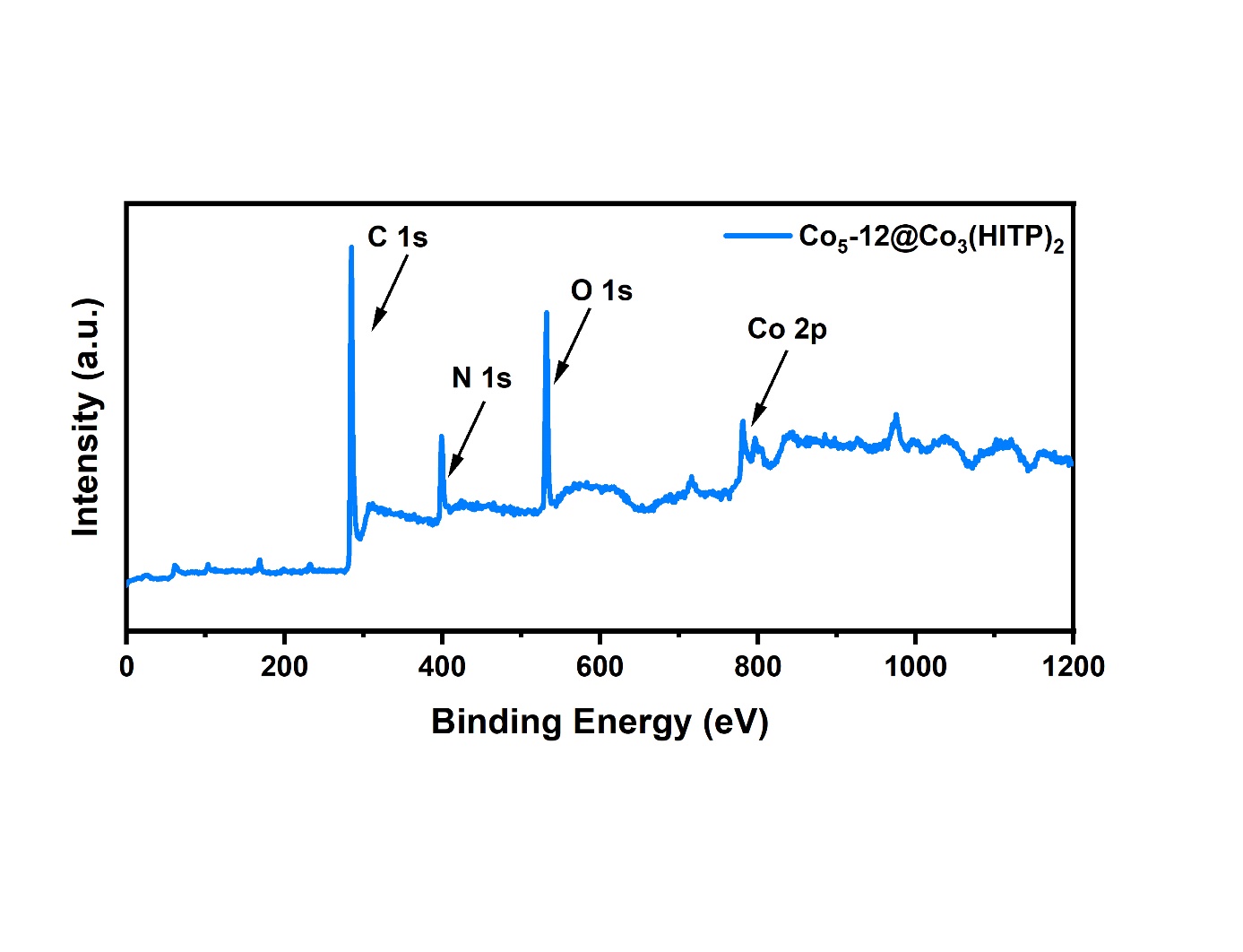


**Figure S3.** XPS survey spectrum of Co_5_-12@Co_3_(HITP)_2_.


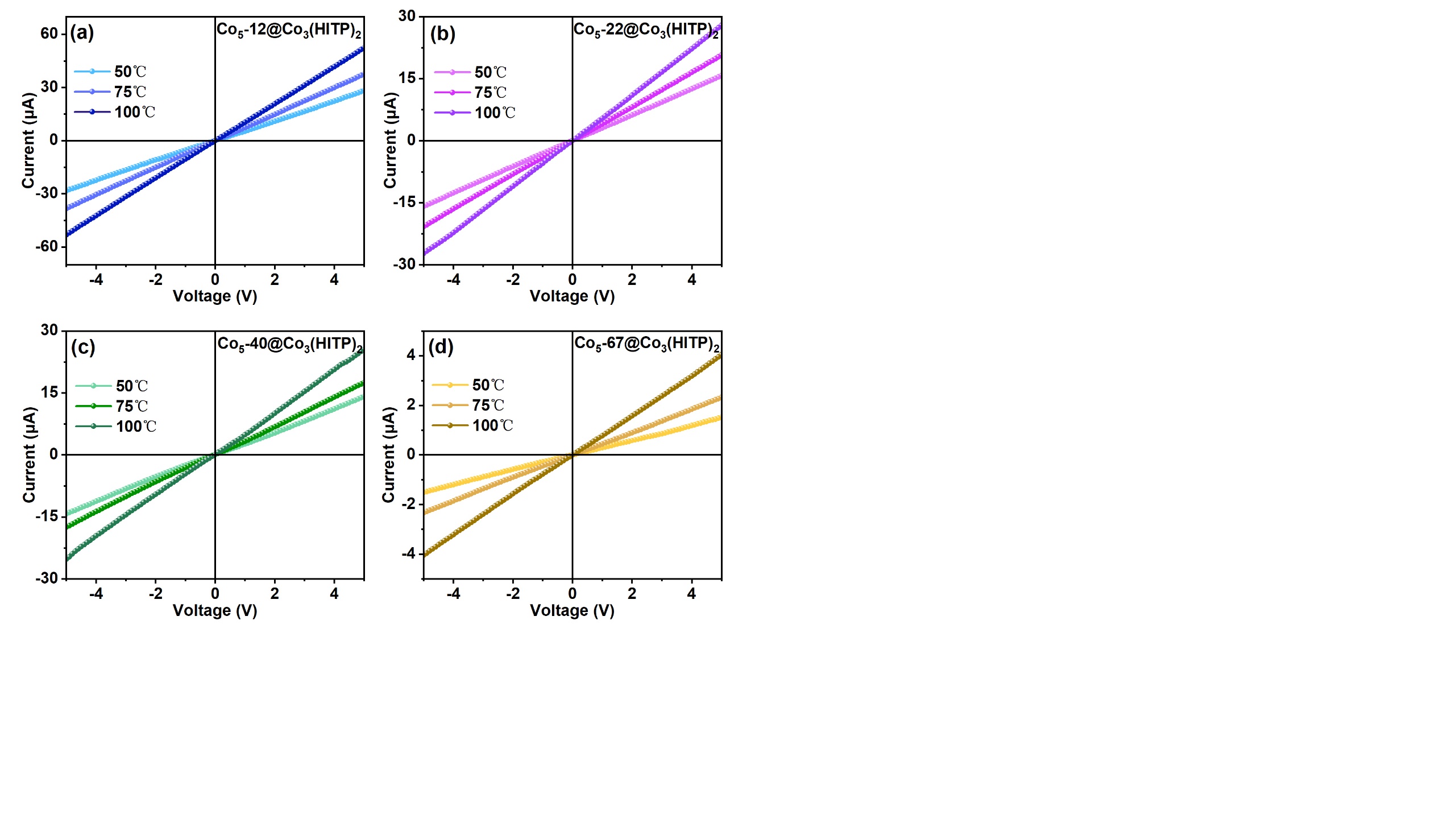


**Figure S4.** I-V curve of Co_5_-*X*@Co_3_(HITP)_2_ sensors: a) Co_5_-12@Co_3_(HITP)_2_, b) Co_5_-22@Co_3_(HITP)_2_$,$ c) Co_5_-40@Co_3_(HITP)_2_ and d) Co_5_-67@Co_3_(HITP)_2_ at different temperature in air atmosphere (two-probe method).


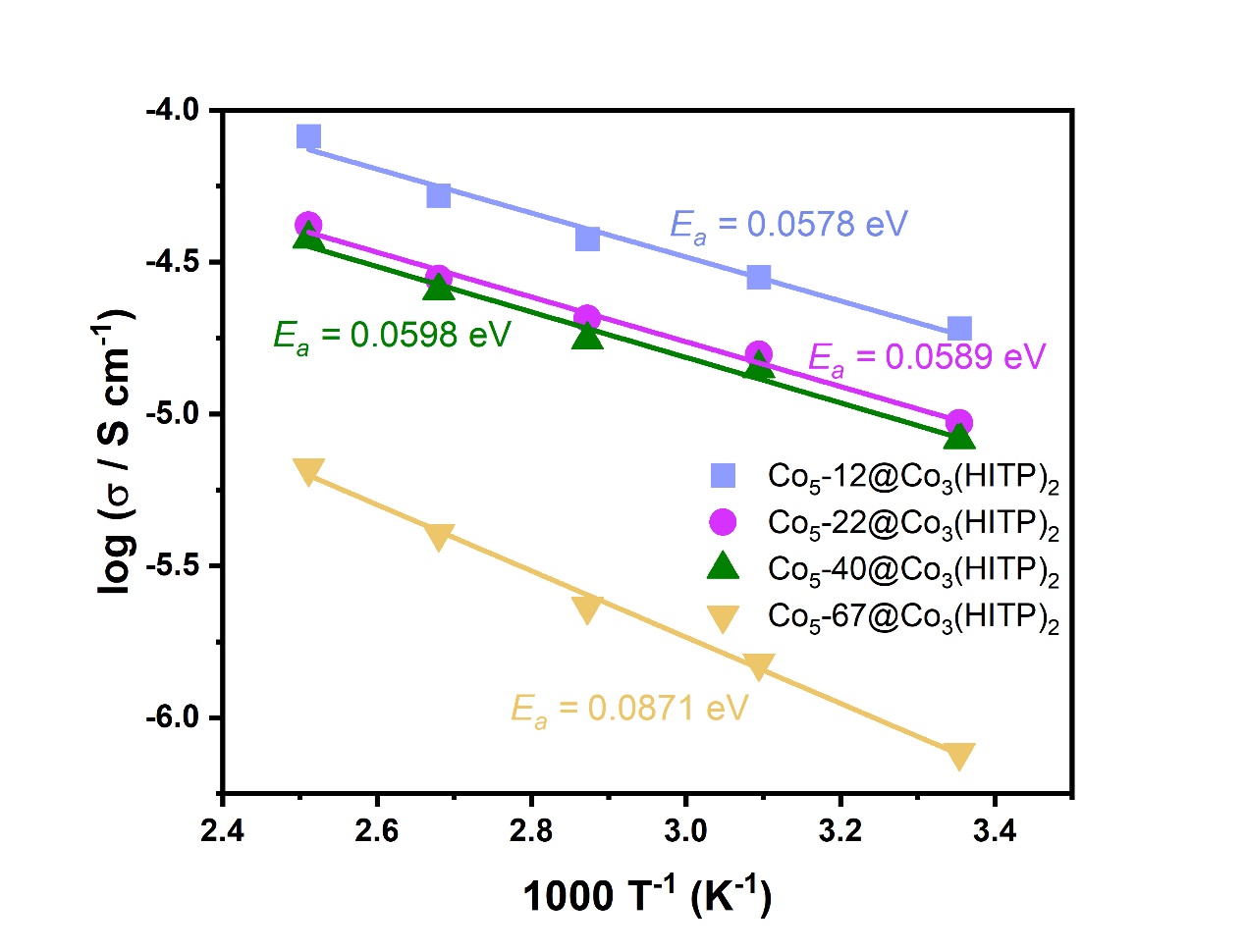


**Figure S5.** Log σ versus T^-1^ plots of Co_5_-*x*@Co_3_(HITP)_2_ sensors.

The conductivity measurements of Co_5_-*x*@Co_3_(HITP)_2_ were tested to investigated the electronic properties (**Figure S4**). The linear and zero drift of the temperature-dependent *I-V* curves indicate good ohmic contact between the Co_5_-*x*@Co_3_(HITP)_2_ films and the Au electrodes (two-prove method). The thermal activation energy was estimated from the linear fitting of log σ versus 1/T (**Figure S5**).^[4]^

**4. H_2_S Gas Sensitivity Testing**


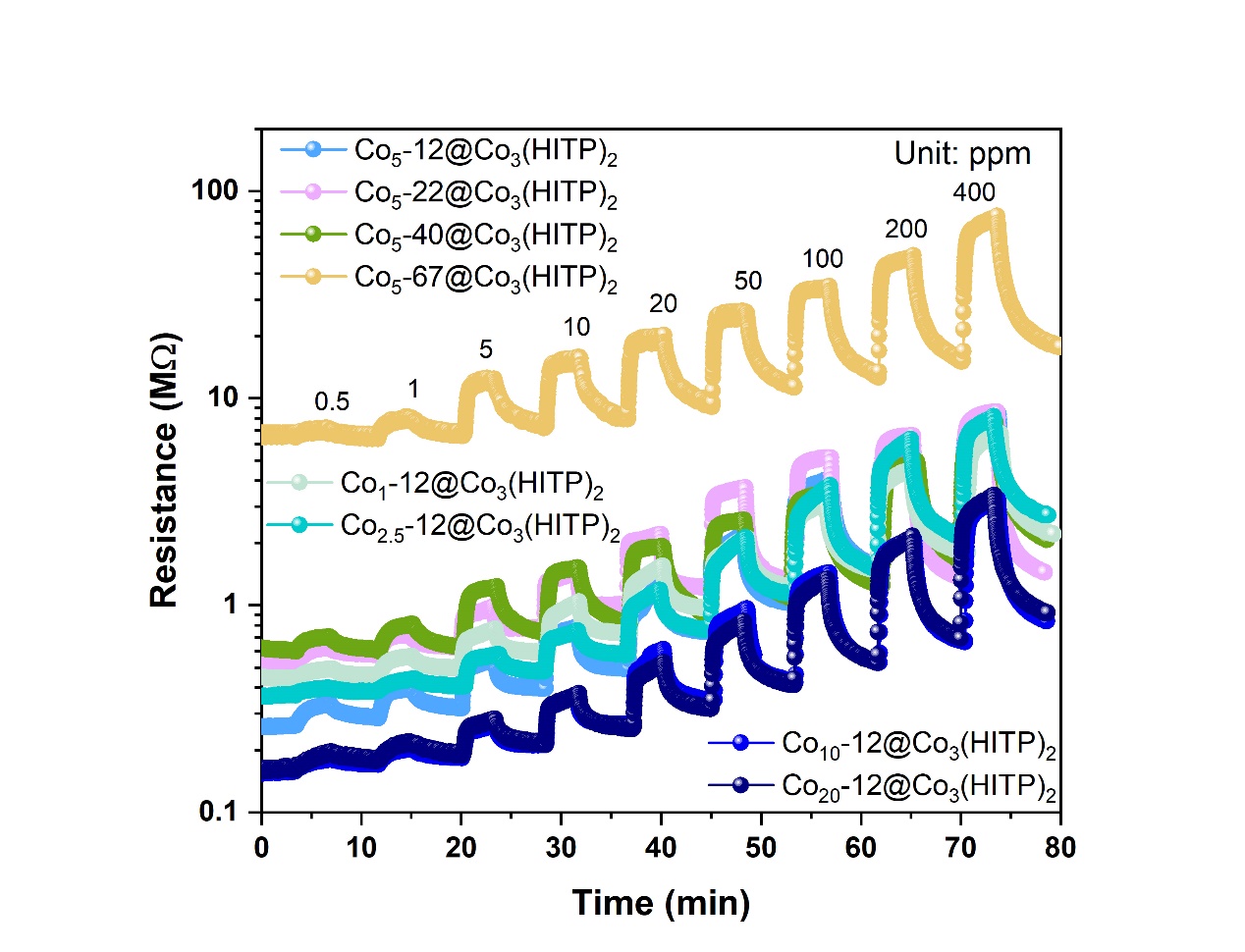


**Figure S6.** The dynamic resistance transients of all sensors toward 0.5–400 ppm H_2_S under 25% RH at RT.

**Figure S6** illustrates the successive resistance curves of all sensors under 25% RH at RT, measured across varying concentrations of H_2_S gas. The results demonstrate that with the rise of concentration, the resistance values of all sensors increased. When exposed to the atmosphere with H_2_S gas, the resistance of all sensors raised and approaching a stable value. While, the recovery performance of all sensors seems to be unsatisfactory. After 9 test cycles, the recovery rates of resistance are 74.1%, 83.2%, 72.9%, 76.1%, 67.4%, 66.7%, 73.9%, and 73.0%, respectively.


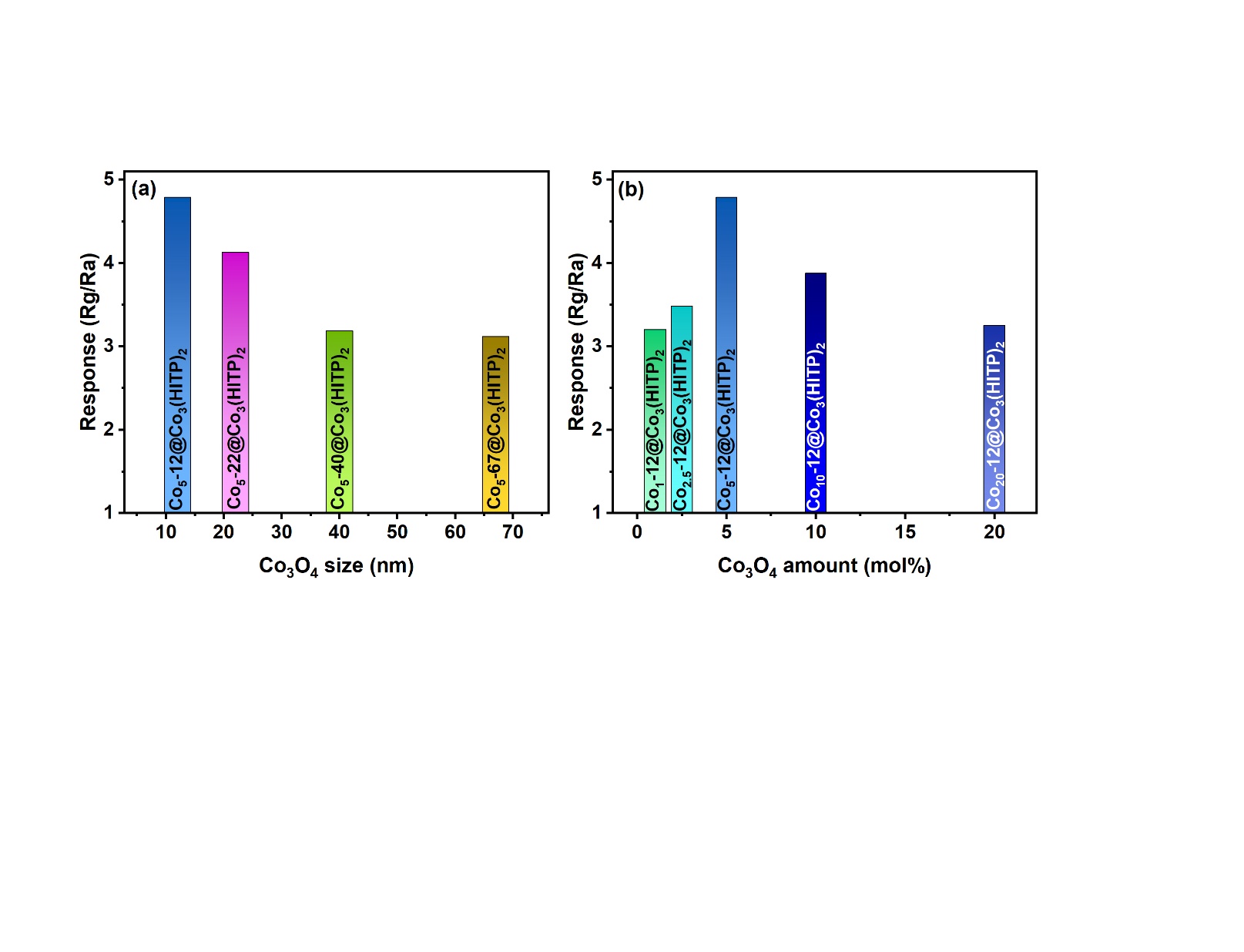


**Figure S7.** Response toward 20 ppm H_2_S changed with Co_3_O_4_ a) size and b) amount.


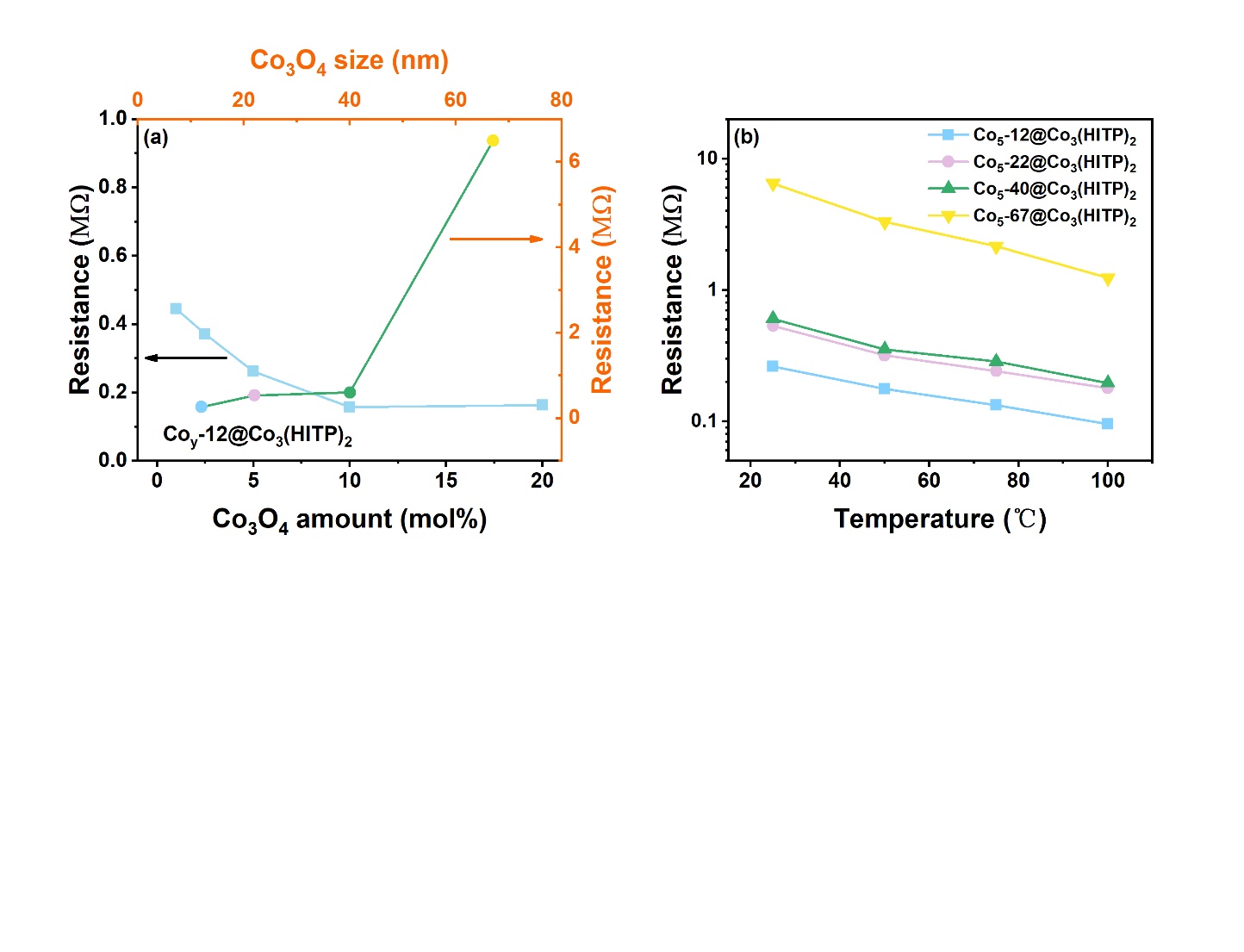


**Figure S8.** a) Electric resistance in air changed with Co_3_O_4_ size and amount. b) Electric resistance in air of Co_5_-*x*@Co_3_(HITP)_2_ (*x*=12, 22, 40, 67) changed with working temperature.

**Figure S8a** shows the baseline resistance changes with Co_3_O_4_ size and amount under 25% RH at RT. It could be found that the resistance increases and decreases with the rise of Co_3_O_4_ size and amount, respectively. **Figure S8b** presents the resistance-temperature characteristics of Co_5_-*x*@Co_3_(HITP)_2_ sensors with varying sizes. Over the temperature range of 25–100℃, the baseline resistance of all sensors decreases markedly with increasing temperature, consistent with the typical semiconducting behavior wherein electrical resistance declines as a function of rising thermal energy.^[5]^


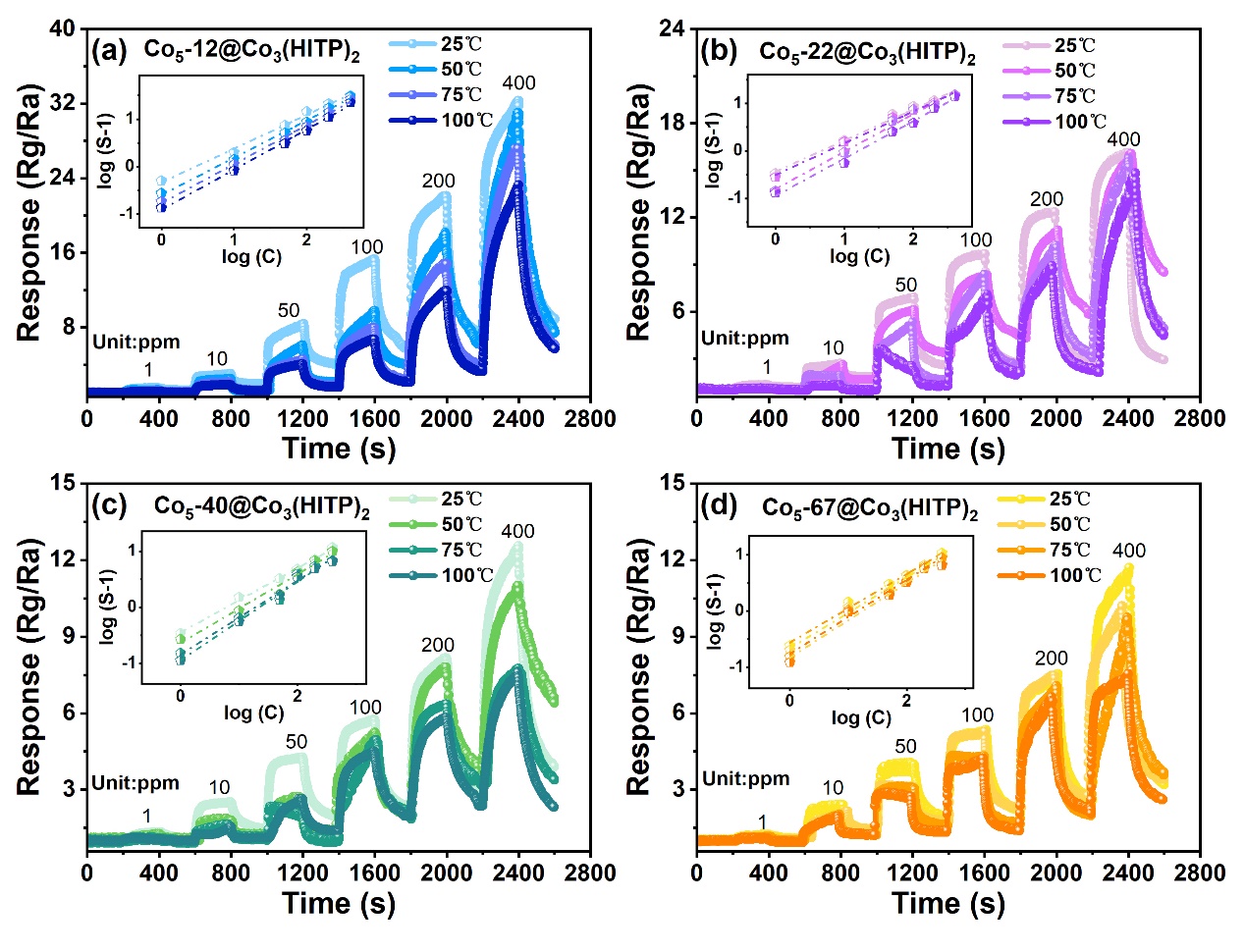


**Figure S9.** a–d) Dynamic response-recovery curves of Co_5_-*x*@Co_3_(HITP)_2_ sensors at different temperatures, insets: the corresponding response-concentration log-log plots.

There is a strong correlation between temperature and electronic resistance of semiconductor sensors (**Figure S8b**), so we studied the impact of temperature on the sensing performance of sensors. We tested the dynamic response-recovery curves of Co_5_-*X*@Co_3_(HITP)_2_ sensors for H_2_S gas ranging from 1 to 400 ppm at 25℃, 50℃, 75℃, and 100℃, as shown in **Figure S9**. It is interesting that the responses of all four sensors exhibit a negative correlation with temperature. As the temperature gradually increases, the response decreases, which might associate with the adsorption-desorption balance of H_2_S. With the raise of temperature, the adsorption and desorption processes on the surface of sensing materials would both enhance, resulting in different gas sensing properties.^[6]^ The test results at room temperature possess the best gas-sensing performance. **Table S2** lists the response values of different sensors at different temperatures when exposed to 100 ppm H_2_S gas. It can be seen that at 100℃, the response values of the four sensors to 100 ppm H_2_S decreased by 56.4%, 34.7%, 21.0%, and 20.8%, respectively, indicating that the increase in temperature leads to a serious decline in sensor performance. The ability to achieve sensitive detection of H_2_S gas at RT is an important advantage of gas sensors based on cMOFs compared to traditional MOS gas sensors.


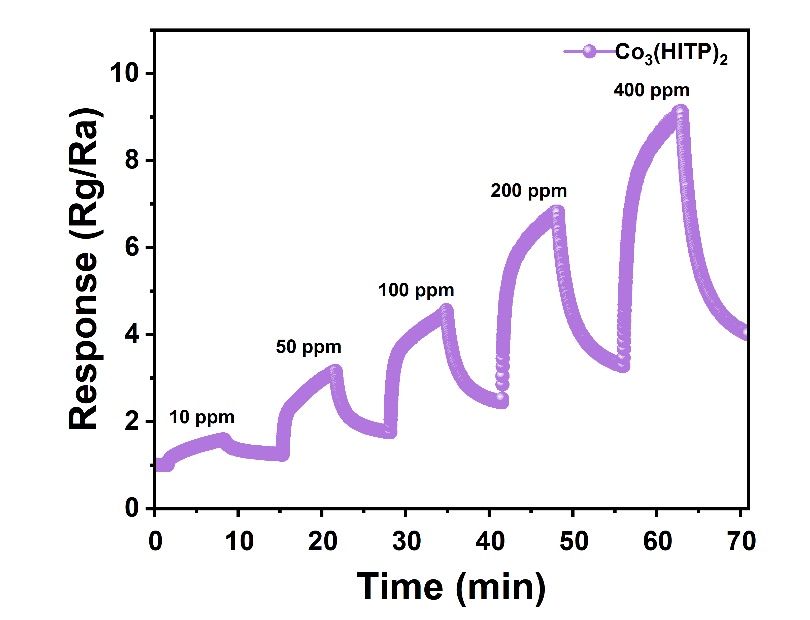


**Figure S10.** Dynamic response-recovery curve of Co_3_(HITP)_2_ toward 10–400 ppm H_2_S under 25% RH at low temperature (0–4℃).


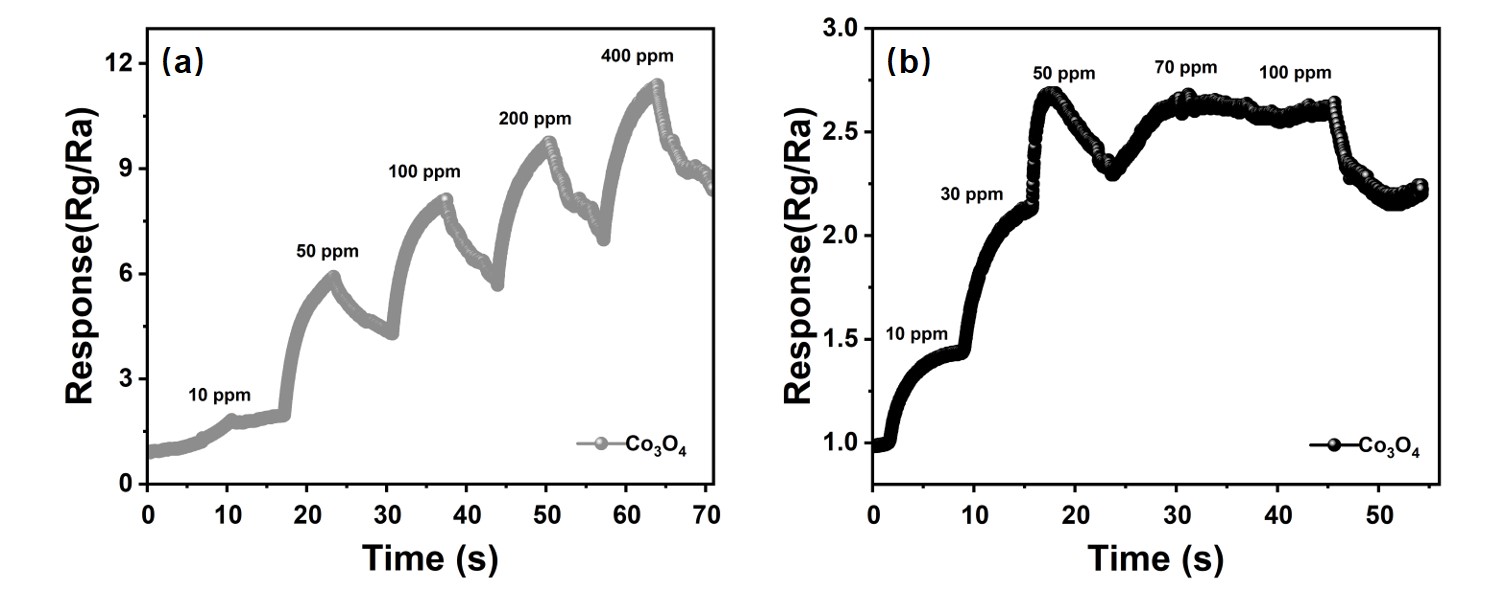


**Figure S11.** Dynamic response-recovery curve of Co_3_O_4_ toward 10–400 ppm H_2_S under 25% RH at a) RT and b) low temperature (0–4℃).


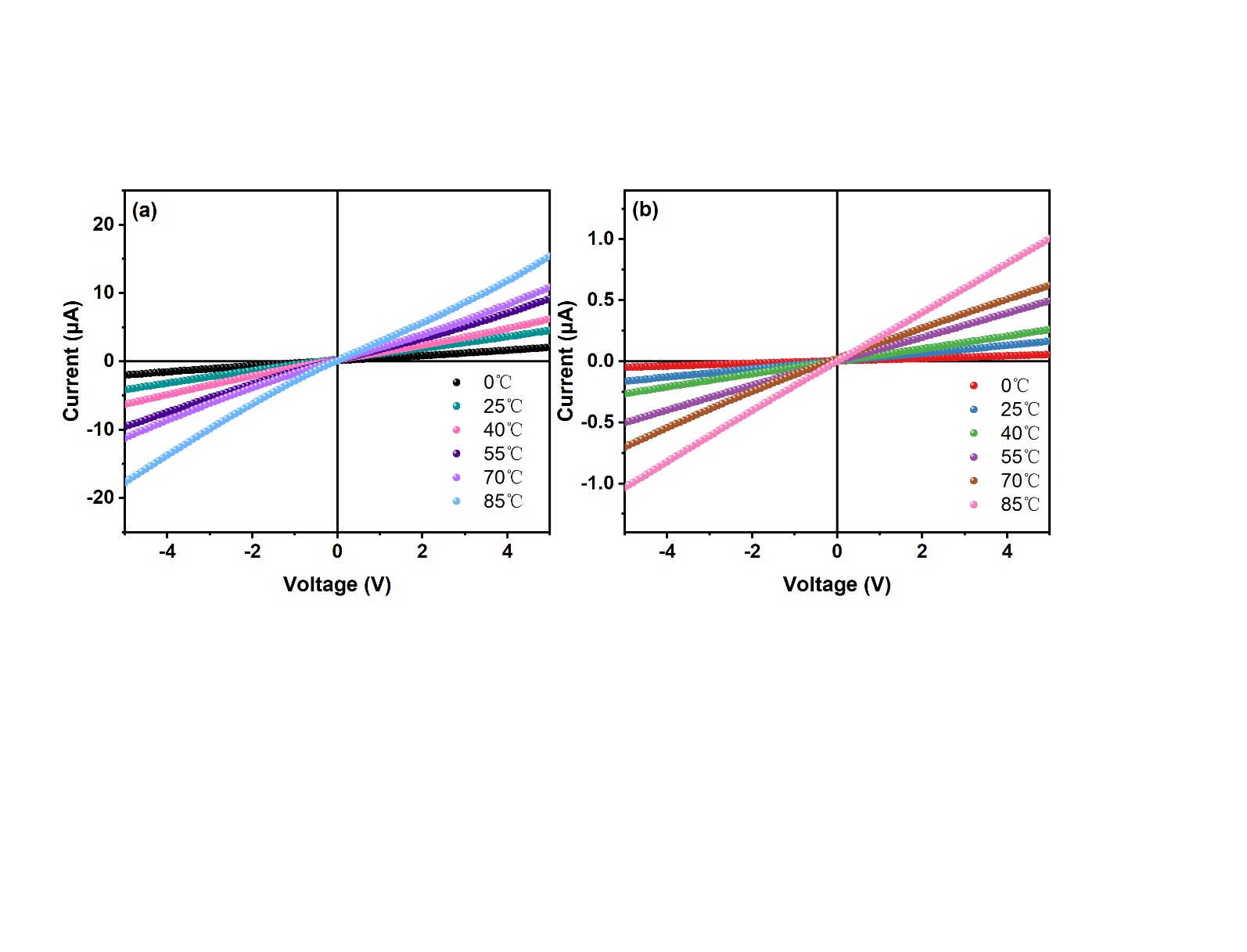


**Figure S12.** I-V curve of a) Co_3_(HITP)_2_ and b) Co_3_O_4_ sensors at different temperature in air atmosphere (two-probe method).


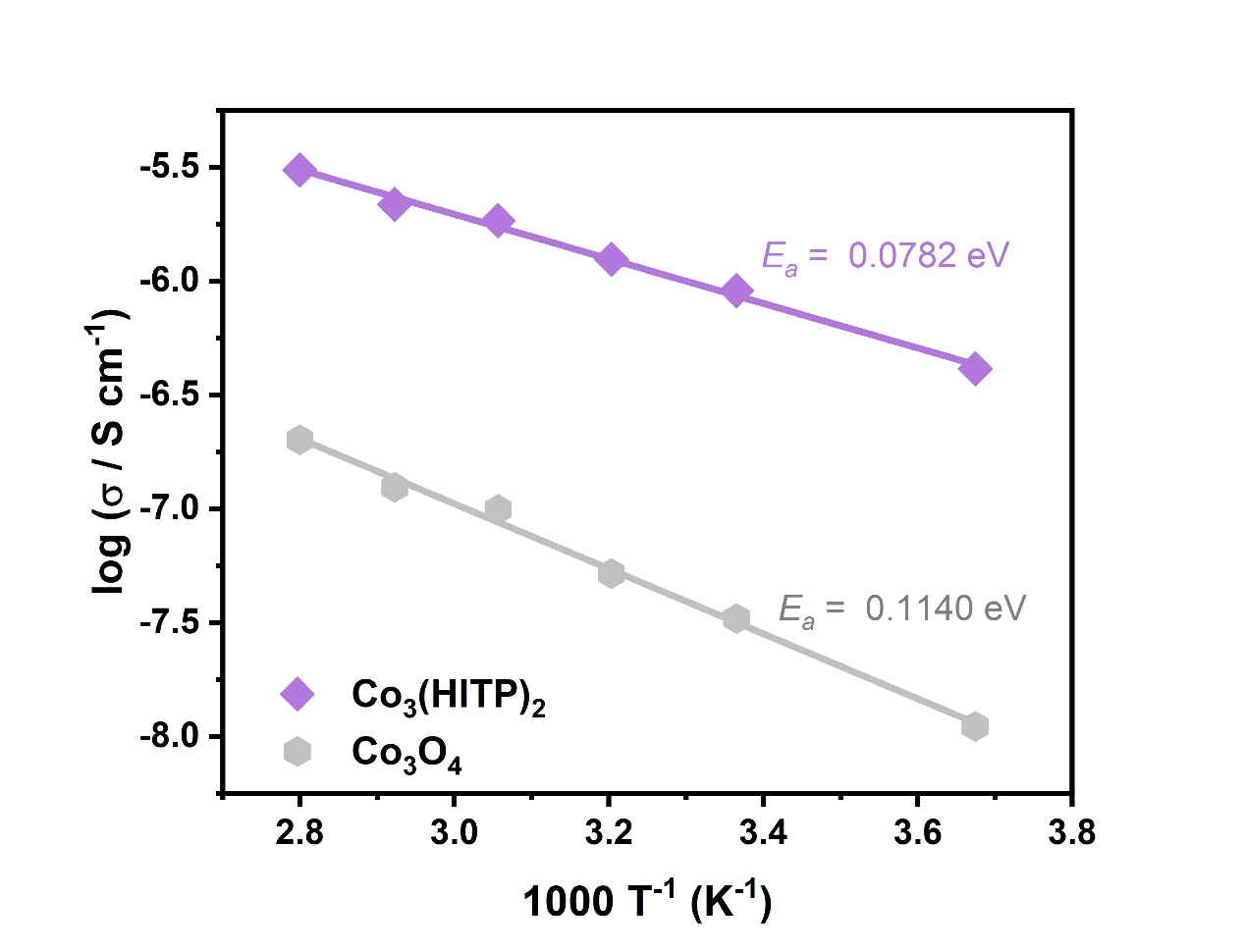


**Figure S13.** Log σ of versus T^-1^ plots of Co_3_(HITP)_2_ and Co_3_O_4_ sensors.

The conductivity measurements of pure Co_3_(HITP)_2_ and Co_3_O_4_ were tested, as shown in **Figure S12**. They also exhibited well linearity and zero drift of the temperature-dependent *I-V* curves. The corresponding thermal activation energy were estimated from the linear fitting of log σ versus 1/T (**Figure S13**).^[4]^


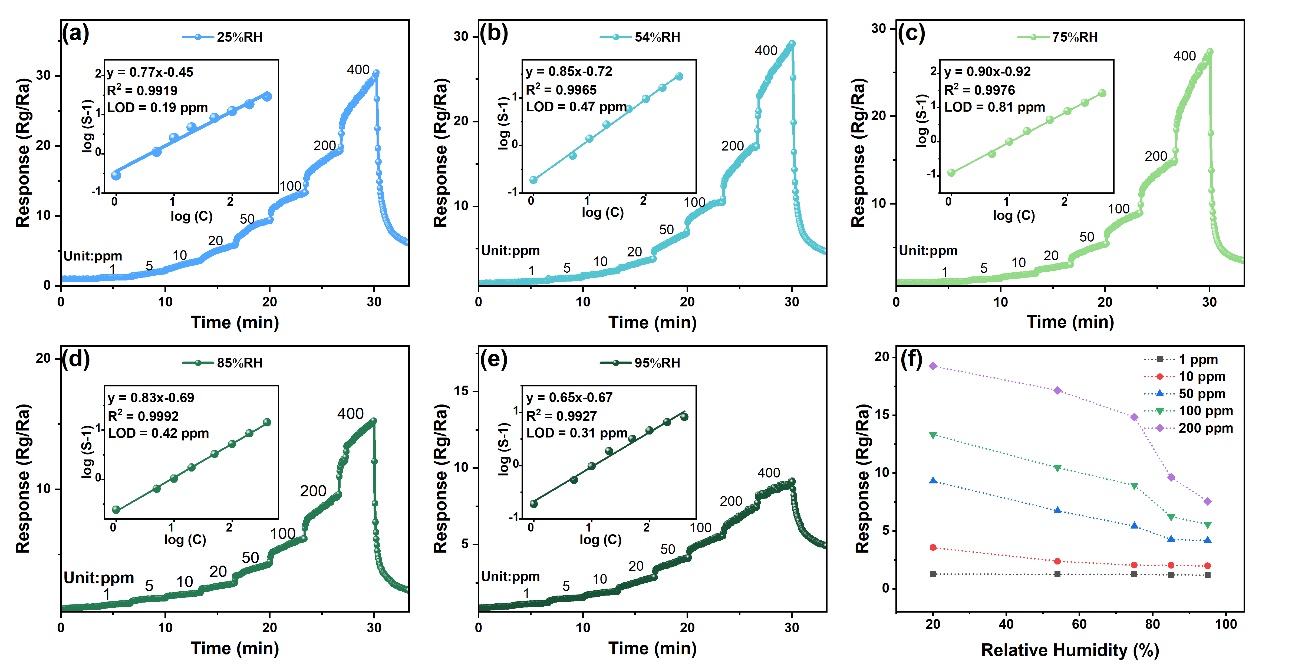


**Figure S14.** a–e) The dynamic response curves of Co_5_-12@Co_3_(HITP)_2_ to 1–400 ppm H_2_S under different RH at RT, insets: response-concentration log-log plots. f) Line chart of response-relative humidity.


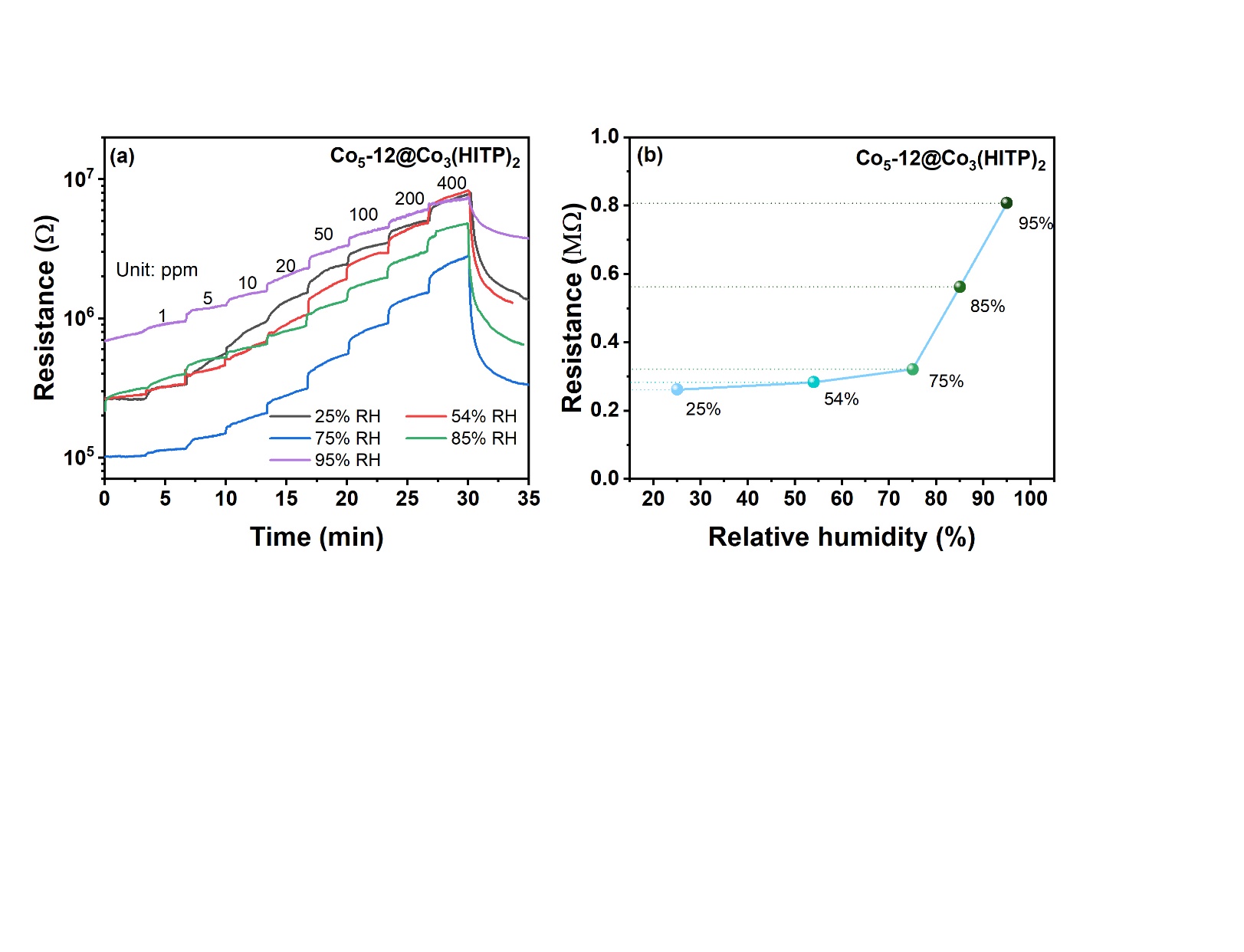


**Figure S15.** a) The dynamic resistance curves of Co_5_-12@Co_3_(HITP)_2_ to 1–400 ppm H_2_S under different RH at RT. b) The resistance in air changed with RH at RT.


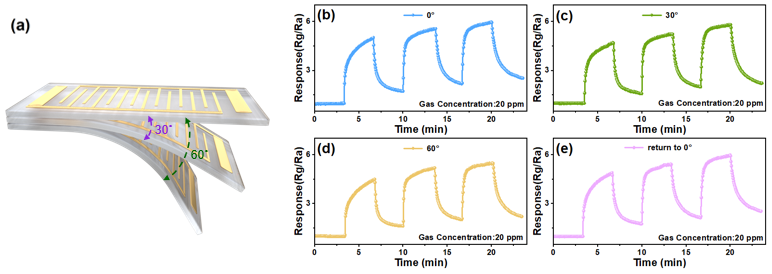


**Figure S16.** a) Lateral view of the flexible electrode. Response of Co_5_-12@Co_3_(HITP)_2_ at different bending angles b) 0°, c) 30°, d) 60°, and e) return to 0°.

As what mentioned before, Co_5_-12@Co_3_(HITP)_2_ sensor based on a flexible substrate, excellent mechanical stability is expected to be one of the most crucial properties for a flexible gas sensor. Therefore, the flexible gas-sensing properties were evaluated under various bending states. **Figure S16a** displays the schematic diagram of a sensor under different bending angles (0°, 30°, 60°, and return to 0°) to illustrate the flexibility. The gas-sensing performance of Co_5_-12@Co_3_(HITP)_2_ sensor to 20 ppm H_2_S at each bending angle is studied, and performed three times to obtain the average response values, as illustrated in **Figure S16b-d**. As can be seen, there is no considerable change in response even after bending the flexible sensor to 60° and return to 0°. In conclusion, the bending characteristics of flexible Co_5_-12@Co_3_(HITP)_2_ sensor illustrate highly flexible nature and good mechanical strength.

**5. Tables**

**Table S1.** The crystallite size (*D_s_*) and the particle size (*P_s_*) of different Co_3_O_4_.

| Sample | $\text{2}\text{θ}\text{ }\text{(°)}$ | $\text{β}\text{ }\text{(°)}$ | *D_s_* (nm) | *P_s_* (nm) |
| --- | --- | --- | --- | --- |
| Co_5_-12@Co_3_(HITP)_2_ | 31.28295 | 0.70 | 13.2 | 12 |
|  | 36.85397 | 0.66 |  |  |
|  | 65.18841 | 0.71 |  |  |
| Co_5_-22@Co_3_(HITP)_2_ | 31.36531 | 0.66 | 14.9 | 22 |
|  | 36.89472 | 0.56 |  |  |
|  | 65.26094 | 0.63 |  |  |
| Co_5_-40@Co_3_(HITP)_2_ | 31.27132 | 0.44 | 21.0 | 40 |
|  | 36.8799 | 0.38 |  |  |
|  | 65.25547 | 0.48 |  |  |
| Co_5_-67@Co_3_(HITP)_2_ | 31.2345 | 0.35 | 23.2 | 67 |
|  | 36.77616 | 0.35 |  |  |
|  | 65.11959 | 0.49 |  |  |

**Table S2.** Response of Co_5_-*x*@Co_3_(HITP)_2_ (*x*=12, 22, 40, 67) to 100 ppm H_2_S.

| Sensors | Response | | | |
| --- | --- | --- | --- | --- |
|  | 25℃ | 50℃ | 75℃ | 100℃ |
| Co_5_-12@Co_3_(HITP)_2_ | 15.50 | 9.71 | 7.93 | 6.67 |
| Co_5_-22@Co_3_(HITP)_2_ | 9.66 | 8.28 | 7.69 | 6.31 |
| Co_5_-40@Co_3_(HITP)_2_ | 5.75 | 5.24 | 4.96 | 4.55 |
| Co_5_-67@Co_3_(HITP)_2_ | 5.35 | 4.88 | 4.33 | 4.24 |

**6. Electric Circuit Design**


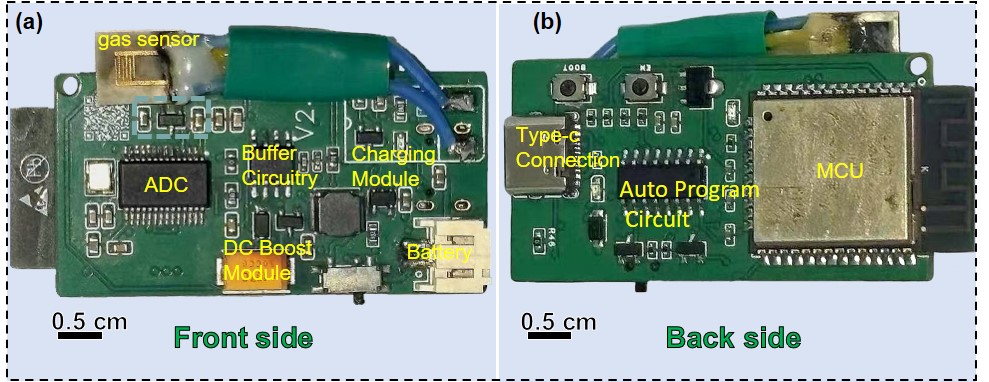


**Figure S17.** a) Front and b) back views of integrated circuit board.


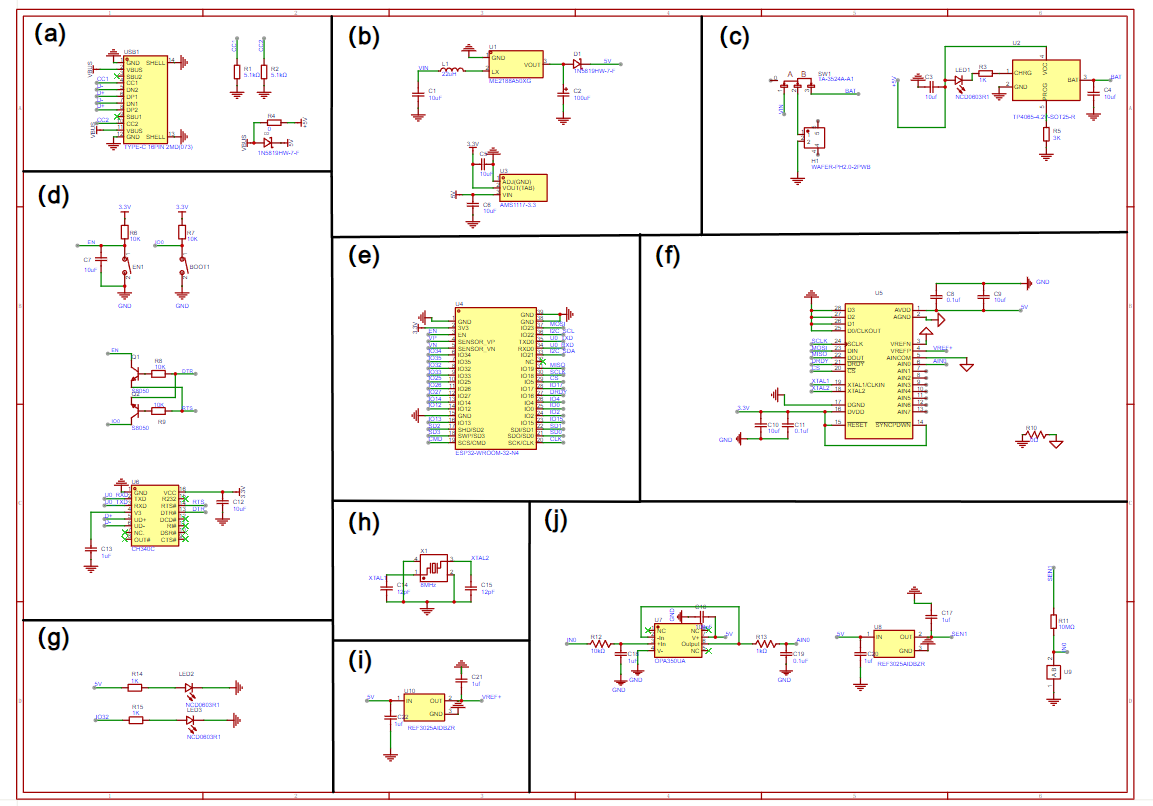


**Figure S18.** a) Type-C interface. b) 5V or 3V voltage supply. c) Switching and charging circuits. d) USB to serial and download circuit. e) The main control chip ESP32-WROOM-32. f) Basic circuit of ads1256. g) The circuit of LEDs. h) The circuit of crystal oscillator. i) Supply reference voltage to ads1256. j) Gas sensor detection and voltage buffer circuit.


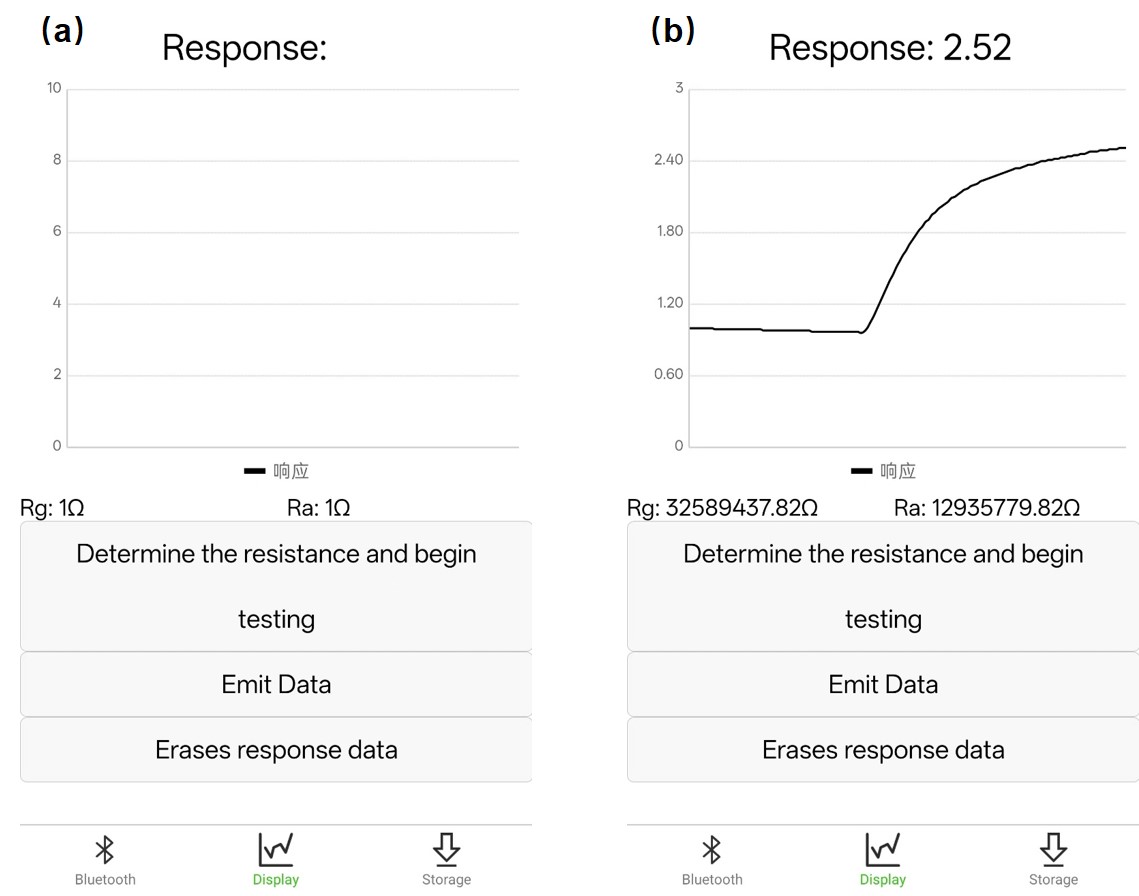


**Figure S19.** The software interface of the mobile phone application: a) before testing and b) when testing.


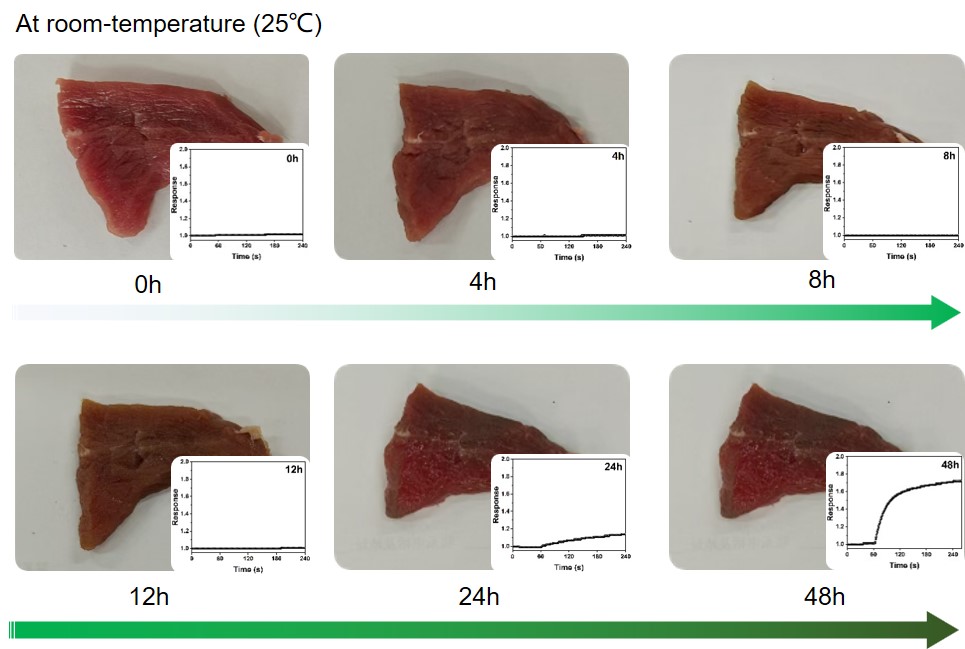


**Figure S20.** The optical images of pork after being stored at room temperature for 0 h, 4 h, 8 h, 12 h, 24 h, and 48 h and the corresponding response.


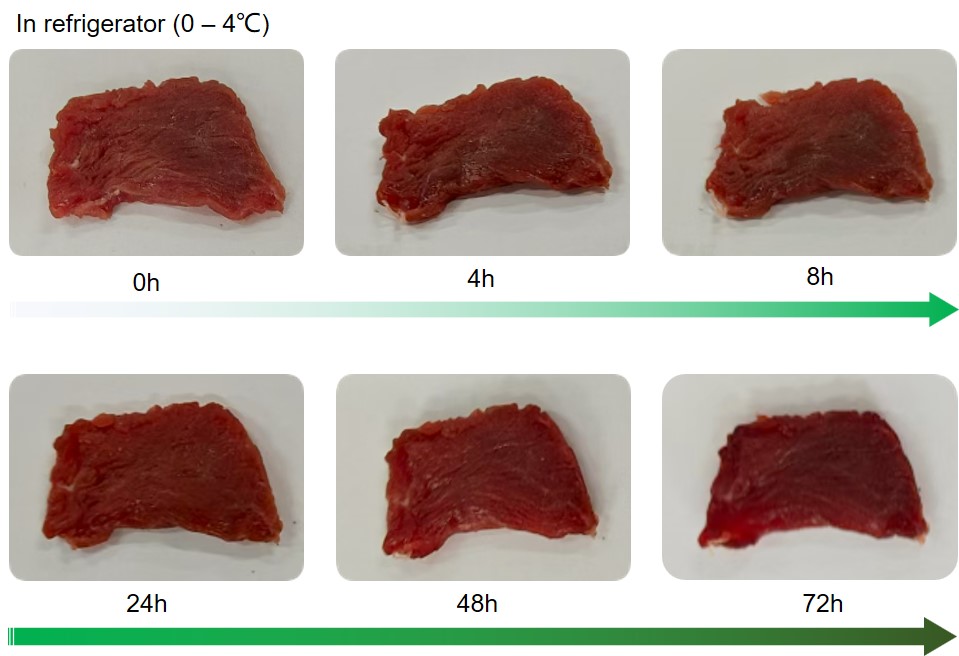


**Figure S21.** The optical images of pork after being stored in refrigerator for 0 h, 4 h, 8 h, 24 h, 48 h, and 72 h and the corresponding response.

**References**

[1] H. Roh, D. Kim, Y. Cho, Y. Jo, J. Alamo, H. Kulik, M. Dincǎ, A. Gumyusenge, Adv. Mater. 2024, 36, 2312382.

[2] Y. Dong, K. He, L. Yin, A. Zhang, Nanotechnology 2007, 18, 435602.

[3] A. Dieguez, A. Ramano-Rodriguez, J. Morante, U. Weimar, M. Schweizer-Berberiich, W. Gopel, Sens. Actuators B 1996, 31, 1–8.

[4] M. Yao, X. Lv, Z. Fu, W. Li, W. Deng, G. Wu, G. Wu, Angew. Chem. Int. Ed. 2017, 56, 16510-16514.

[5] S. Cai, X. Song, Z. Chi, Y. Fu, Z. Fang, S. Geng, Y. Kang, X. Yang, J. Qin, W. Xie, Sens. Actuators B-Chem. 2021, 343, 130118.

[6] G. Korotcenkov, M. Ivanov, I. Blinov, J. Stetter, Thin Solid Films 2007, 515, 3987–3996.
